# Yin Yang 1‐Induced Long Noncoding RNA DUXAP9 Drives the Progression of Oral Squamous Cell Carcinoma by Blocking CDK1‐Mediated EZH2 Degradation

**DOI:** 10.1002/advs.202207549

**Published:** 2023-07-03

**Authors:** Wenkai Zhou, Yisheng Feng, Chengzhong Lin, Chi Kuan CHAO, Ziqi He, Shiyao Zhao, Jieyuan Xue, Xu‐Yun Zhao, Wei Cao

**Affiliations:** ^1^ Department of Oral and Maxillofacial & Head and Neck Oncology Shanghai Ninth People's Hospital College of Stomatology Shanghai Jiao Tong University School of Medicine Shanghai 200011 China; ^2^ National Center for Stomatology National Clinical Research Center for Oral Diseases Shanghai Key Laboratory of Stomatology Shanghai 200011 China; ^3^ The 2nd Dental Center Shanghai Ninth People's Hospital College of Stomatology Shanghai Jiao Tong University School of Medicine College of Stomatology Shanghai Jiao Tong University Shanghai 200011 China; ^4^ Department of Cardiology Shanghai Chest Hospital Shanghai Jiao Tong University Shanghai 200030 China; ^5^ Department of Biochemistry and Molecular Cell Biology Shanghai Key Laboratory for Tumor Microenvironment and Inflammation Key Laboratory of Cell Differentiation and Apoptosis of the Chinese Ministry of Education Shanghai Jiao Tong University School of Medicine Shanghai 200025 China

**Keywords:** EZH2, LncRNA DUXAP9, oral squamous cell carcinoma, ubiquitination, Yin Yang 1 (YY1)

## Abstract

LncRNAs play a critical role in oral squamous cell carcinoma (OSCC) progression. However, the function and detailed molecular mechanism of most lncRNAs in OSCC are not fully understood. Here, a novel nuclear‐localized lncRNA, DUXAP9 (DUXAP9), that is highly expressed in OSCC is identified. A high level of DUXAP9 is positively associated with lymph node metastasis, poor pathological differentiation, advanced clinical stage, worse overall survival, and worse disease‐specific survival in OSCC patients. Overexpression of DUXAP9 significantly promotes OSCC cell proliferation, migration, invasion, and xenograft tumor growth and metastasis, and upregulates N‐cadherin, Vimentin, Ki67, PCNA, and EZH2 expression and downregulates E‐cadherin in vitro and in vivo, whereas knockdown of DUXAP9 remarkably suppresses OSCC cell proliferation, migration, invasion, and xenograft tumor growth in vitro and in vivo in an EZH2‐dependent manner. Yin Yang 1 (YY1) is found to activate the transcriptional expression of DUXAP9 in OSCC. Furthermore, DUXAP9 physically interacts with EZH2 and inhibits EZH2 degradation via the suppression of EZH2 phosphorylation, thereby blocking EZH2 translocation from the nucleus to the cytoplasm. Thus, DUXAP9 can serve as a promising target for OSCC therapy.

## Graphical abstract

The authors identify a lncRNA DUXAP9 is highly correlated with poor clinicopathological features in oral squamous cell carcinoma (OSCC) patients. YY1 transcriptionally induces DUXAP9 expression, which drives OSCC pathogenesis in an EZH2‐dependent manner. Mechanistically, DUXAP9 enhances EZH2 stability by blocking the phosphorylation of EZH2 by CDK1 and inhibiting its translocation from the nucleus to the cytoplasm for degradation.

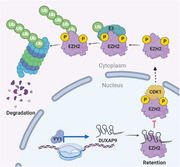

## 1 Introduction

Oral squamous cell carcinoma (OSCC) is one of the most common malignancies of the head and neck region in the world and is widely represented as a heterogeneous tumor with more aggressive phenotypes.^[^ ^1^ ^]^ Although surgical resection followed by postoperative radiotherapy and/or chemotherapy has provided considerable therapeutic options, the 5 year overall survival rate of OSCC patients remains poor due to the complexity and uncertainty of OSCC pathogenesis. Although a series of hotspot mutational genes, such as tumor protein p53 (TP53), cyclin‐dependent kinase inhibitor 2A (CDKN2A), phosphatase and tensin homolog (PTEN), phosphatidylinositol‐4,5‐bisphosphate 3‐kinase catalytic subunit alpha (PIK3CA), and notch receptor 1 (NOTCH1), have been identified,^[^ ^2^ ^]^ it is still far from understanding the pathogenesis of OSCC. Recently, epigenetic alterations such as posttranslational modifications of histones and long noncoding RNAs (lncRNAs) were proven to be critical regulators that mediate OSCC tumorigenesis.^[^ ^3^ ^]^

Enhancer of zeste homolog 2 (EZH2) is the catalytic subunit of Polycomb repressive complex 2 (PRC2), a highly conserved histone methyltransferase that methylates lysine‐27 of histone H3 (H3‐K27), which has been largely characterized to function as a suppressor of gene transcriptional regulation, implicated in cell differentiation, maintenance of adult stem cell populations, and tumor development.^[^ ^4^ ^]^

In our previous studies, EZH2 was identified as a key oncogenic driver that promotes the malignant transformation of oral leukoplakia. We demonstrated that EZH2 expression was remarkably elevated in OSCC specimens and cell lines, and its aberrant expression was associated with poor differentiation, histologic signs of the severity of head and neck squamous cell carcinoma (HNSCC), and poorer overall survival and disease‐free survival, which indicated that EZH2 is an independent prognostic indicator for patients with OSCC^.[^ ^5^ ^]^ EZH2 was also confirmed to be involved in the progression of upper digestive tract tumors and is closely associated with poor prognosis of those patients.^[^ ^5^, ^6^ ^]^ In addition, a gambogenic acid (GNA) derivative targeting EZH2 was screened, which triggers EZH2 degradation through CHIP‐mediated ubiquitination.^[^ ^7^ ^]^ However, the potential mechanisms by which lncRNAs post‐translationally regulate EZH2 in OSCC progression remain to be fully elucidated and further investigated.

Long noncoding RNAs (lncRNAs) belong to a subgroup of noncoding RNAs that are at least 200 nucleotides, including antisense, intergenic, and intronic transcripts and pseudogenes.^[^ ^8^ ^]^ The transcription, processing and metabolism of lncRNAs are regulated differently from those of protein‐coding genes.^[^ ^8a^ ^]^ LncRNAs are localized both in the nucleus and cytoplasm, and the specific localization of these lncRNAs determines their regulatory mechanisms in gene expression and biological functions such as chromatin modification, interaction with transcriptional factors, mRNA processing, acting as “molecular sponge” and creating ribonucleoprotein complexes.^[^ ^9^ ^]^ In our prior studies, decreased expression of lncRNA PTENP1 was found to promote malignant behaviors, and its reduced expression was associated with the poor survival rate of patients with HNSCC.^[^ ^10^ ^]^ Furthermore, a three‐lncRNA signature was identified as a novel biomarker panel for the accurate prognosis prediction of patients with OSCC.^[^ ^11^ ^]^ Additionally, lncRNA RC3H2 was found to facilitate the proliferation and invasion of OSCC cells by sponging with miR‐101‐3p to release the expression of EZH2,^[^ ^12^ ^]^ suggesting that lncRNAs play a vital role as predictive biomarkers or therapeutic targets in OSCC pathogenesis.

Here, we identified a novel lncRNA, DUXAP9 (ENSG00000225210), which is dramatically upregulated in OSCC tissue and closely associated with the survival rate of OSCC patients. Interestingly, a recent report showed that DUXAP9 promotes hepatocellular carcinoma cell stemness by directly binding to the 3′ UTR of Sox9 and enhancing its mRNA stability.^[^ ^13^ ^]^ The N6‐methyladenosine modification of DUXAP9 promotes renal cancer cell proliferation and motility by activating the PI3K/AKT signaling pathway.^[^ ^14^ ^]^ Furthermore, DUXAP9 promotes nonsmall cell lung cancer cell proliferation and metastasis by interacting with Cbl‐b to prevent the degradation of EGFR and thus ensures the activation of EGFR signaling.^[^ ^15^ ^]^ In our study, high expression of DUXAP9 was detected to be localized in the nucleus of OSCC cells and tissues by qRT‐PCR and in situ FISH. Overexpression of DUXAP9 was frequently observed in moderate/poor pathological grade, advanced clinical stage, lymph node metastasis, worse overall survival (OS), and worse disease‐specific survival (DSS). Yin Yang 1 (YY1) was verified to contribute to the overexpression of DUXAP9 in OSCCs. Loss‐of‐function and gain‐of‐function assays showed that DUXAP9 promotes the proliferation, migration, invasion, and metastasis of OSCC cells in vitro and in vivo. Mechanistically, DUXAP9 was revealed to enhance the stability of the EZH2 protein by inhibiting cyclin‐dependent kinase 1‐mediated EZH2 phosphorylation and ubiquitination by various mutational assays.

## 2 Results

### 2.1 DUXAP9 is a nuclear‐localized lncRNA that is highly expressed in OSCC and correlated with poor clinicopathological features in OSCC patients

**Figure 1:**
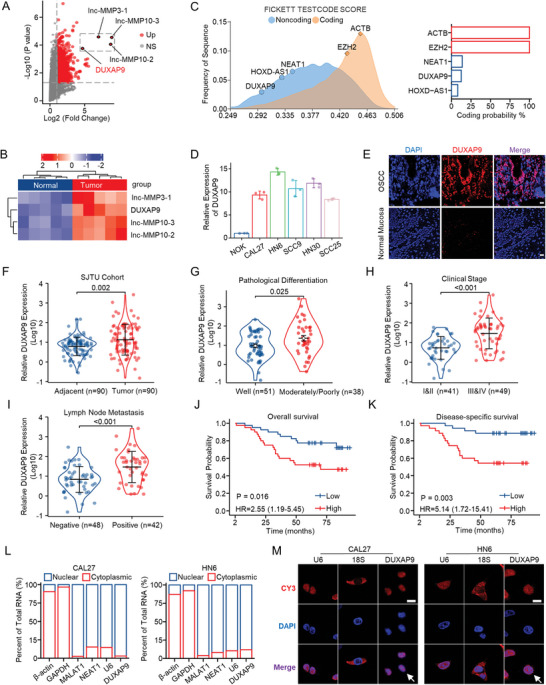
DUXAP9 is a nuclear lncRNA that is highly expressed in OSCC patients and positively correlated with poor clinicopathologic features. A) Volcano plot used to visualize the differential lncRNAs from the microarray assay compared between tumor and adjacent normal tissues of OSCC patients. Four lncRNAs that are highly expressed in tumor samples of OSCC patients are highlighted. B) Heatmap of highlighted lncRNAs in tumor and adjacent normal tissues of OSCC patients. C) DUXAP9 coding potential was predicted and visualized using CPC2. D) The expression of DUXAP9 in 5 OSCC cell lines and normal oral epithelial cells (NOK) was measured by qRT‐PCR. E) The expression of DUXAP9 was detected using an RNAscope assay in OSCC tissues and adjacent normal tissues. Scale bars, 20 µm. F) The expression of DUXAP9 in OSCC tissues (Tumor) and adjacent normal tissues (Adjacent) was measured by qRT‐PCR. Data were calculated by two‐tailed unpaired Student's *t*‐test. G–I) The expression of DUXAP9 in OSCC tissues classified according to pathological differentiation G), clinical stage H), and lymph node metastasis I). Data in (G–I) were calculated by the Wilcoxon rank‐sum test. J,K) Overall survival (OS) J) and disease‐specific survival (DSS) K) of OSCC patients with low and high DUXAP9 were analyzed by Kaplan–Meier plots. *n* = 80, *p* values were determined by a two‐tailed log‐rank test. L) The cellular distribution of DUXAP9 was shown by cell nuclear/cytoplasmic fractionation and qRT‐PCR in CAL27 and HN6 cells. M) FISH analysis of DUXAP9 in CAL27 and HN6 cells. Scale bar, 10 µm. Data are presented as the mean ± SD from three independent experiments.

Recently, large amounts of evidence have implied that the aberrant expression and binding of lncRNAs with key nuclear factors reprogram gene expression, which serves as a critical mechanism that drives cancer progression. To identify abnormally upregulated lncRNAs in oral squamous cell carcinoma (OSCC), we analyzed a lncRNA microarray dataset that was performed in five paired OSCCs and the corresponding adjacent normal tissues.^[^ ^16^ ^]^ The results showed that 535 of the 65 437 lncRNAs were significantly increased in OSCC (**Figure** **1**A). The clinical characteristics of the five OSCC patients used for the microarray assay and bioinformatics analysis are shown in Table S1 (Supporting Information). Among the significantly increased lncRNAs, four lncRNAs, Inc‐MMP3‐1, Inc‐MMP10‐2, Inc‐MMP10‐3, and DUXAP9, were the most highly expressed lncRNAs, but only DUXAP9 was a validated lncRNA in NCBI GenBank (Gene ID: 503 638) (Figure 1B). Using Coding Protein Calculator 2.0 (http://cpc.2.gao‐lab.org), we predicted that DUXAP9 lacks coding capacity compared with known noncoding genes (NEAT1 and HOXD‐AS1) and coding genes (EZH2 and ACTB) (Figure 1C).

To verify DUXAP9 expression in cancer, we performed a pancancer analysis that compared tumor tissues and paracancerous tissues using The Cancer Genome Atlas (TCGA) database. Notably, DUXAP9 is upregulated in most cancers (Figure S1A, Supporting Information), including head and neck squamous cell carcinoma (HNSCC), in the TCGA database. Importantly, the expression of DUXAP9 was upregulated in 5 OSCC cell lines compared with normal oral epithelial cells (Figure 1D). The RNAscope assay also showed that DUXAP9 is enriched in OSCC tumor tissues (Figure 1E; and Figure S1B, Supporting Information). These results suggest that DUXAP9 may play an oncogenic role in the progression and development of OSCC. To investigate DUXAP9 expression in OSCC, we measured DUXAP9 expression by qRT‐PCR in 90 paired OSCC samples from the Shanghai Jiao Tong University (SJTU) cohort compared with paired adjacent normal tissues. The results clearly demonstrated that DUXAP9 expression in OSCC tissues is higher than that in adjacent normal tissues (Figure 1F; and Figure S1C, Supporting Information). The expression of DUXAP9 is also higher in genotype‐tissue expressed (GTEx) OSCC tumor samples from TCGA than in their corresponding paracancerous controls (Figure S1D, S1E, Supporting Information). Furthermore, we examined the clinical relevance of DUXAP9 in OSCCs. We revealed that DUXAP9 expression is significantly higher in the paired OSCC samples from the SJTU cohort, which had moderate/poor pathological differentiation (Figure 1G), advanced clinical stage (Figure 1H), and lymph node metastasis (Figure 1I). Moreover, Kaplan–Meier survival curve analysis (K–M analysis) indicated that high DUXAP9 expression was associated with poor overall survival (OS) and disease‐specific survival (DSS) in patients from the SJTU cohort (Figure 1J,K) and TCGA cohort using Gene Expression Profiling Interactive Analysis (GEPIA) (Figure S1F, Supporting Information).

Next, to determine the subcellular localization of DUXAP9, we performed nuclear and cytoplasmic fractionation using CAL27 and HN6 cells, which were randomly selected from OSCC cell lines with the highest and intermediate DUXAP9 expression levels compared to NOK cells, followed by qRT‐qPCR. The results indicate that the majority of DUXAP9 transcripts are in the nucleus (Figure 1L). Confocal microscopy of fluorescence in situ hybridization (FISH) analysis also demonstrated that DUXAP9 is primarily expressed in the nucleus of CAL27 and HN6 cells (Figure 1M). These results suggest that DUXAP9 may exert its biological function in the nucleus.

### 2.2 DUXAP9 promotes OSCC cell proliferation, invasion, and metastasis in vitro and in vivo

Since DUXAP9 transcripts are mainly localized in the nucleus, siRNA transfection was reported to be less efficient than antisense oligonucleotides (ASOs) for modulating nuclear‐localized lncRNAs.^[^ ^17^ ^]^ To improve the silencing efficiency of DUXAP9, we used a Smart Silencer containing a mix of 3 independent siRNAs and 3 independent ASOs targeting DUXAP9 (SS‐DUXAP9) to knock down DUXAP9 expression in CAL27 and HN6 cells (Figure S2A and Table S2, Supporting Information).

**Figure 2:**
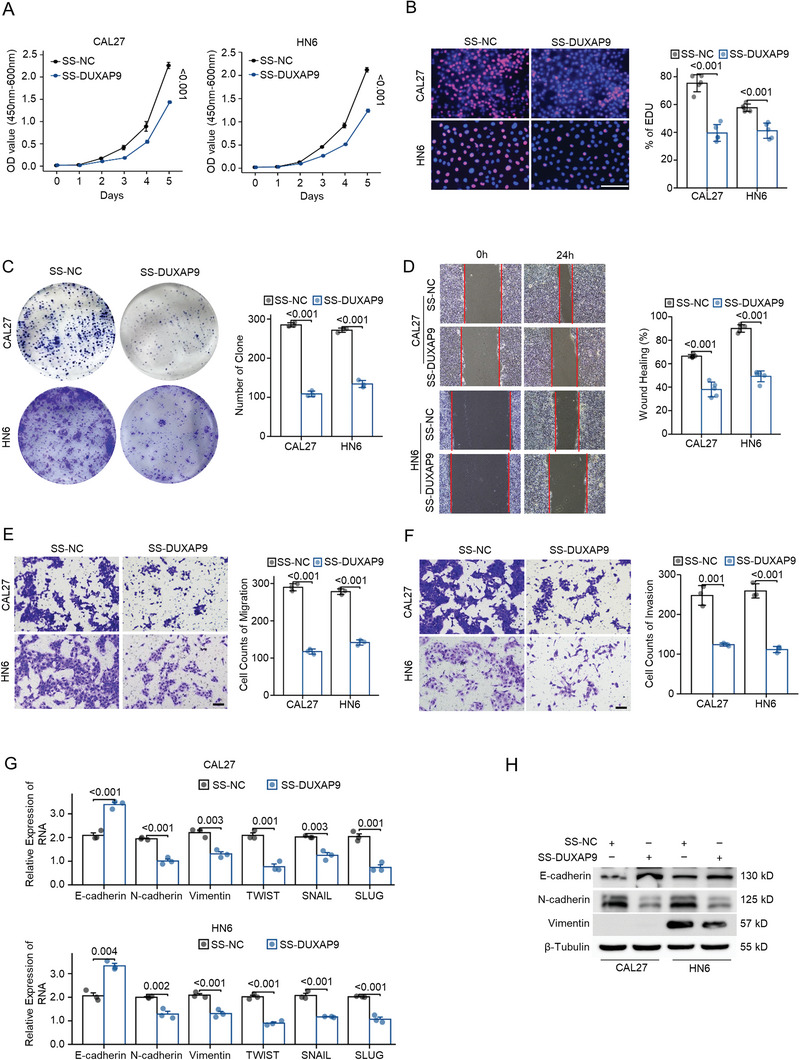
Knockdown of DUXAP9 inhibits OSCC cell growth, migration, and invasion in vitro. A–C) The proliferation of CAL27 and HN6 cells transfected with DUXAP9 knockdown (SS‐DUXAP9) or control (SS‐NC) ASO was evaluated by CCK‐8 assays A), EdU assays B), and colony formation assays C). Scale bars, 100 µm. D,E) The migration behavior of CAL27 and HN6 cells transfected with SS‐DUXAP9 or SS‐NC ASO was assessed by wound healing assays D) and transwell assays E). Scale bars, 100 µm. F) The invasion ability of CAL27 and HN6 cells transfected with SS‐DUXAP9 or SS‐NC ASO was determined by Transwell assays. Scale bars, 100 µm. G,H) The expression of EMT markers as indicated was measured by qRT‐PCR G) and western blot analysis H) in SS‐DUXAP9‐ and SS‐NC ASO‐transfected OSCC cells. Data are presented as the mean ± SD from three independent experiments. Data in (A–G) were calculated by two‐tailed unpaired Student's *t*‐test.

**Figure 3:**
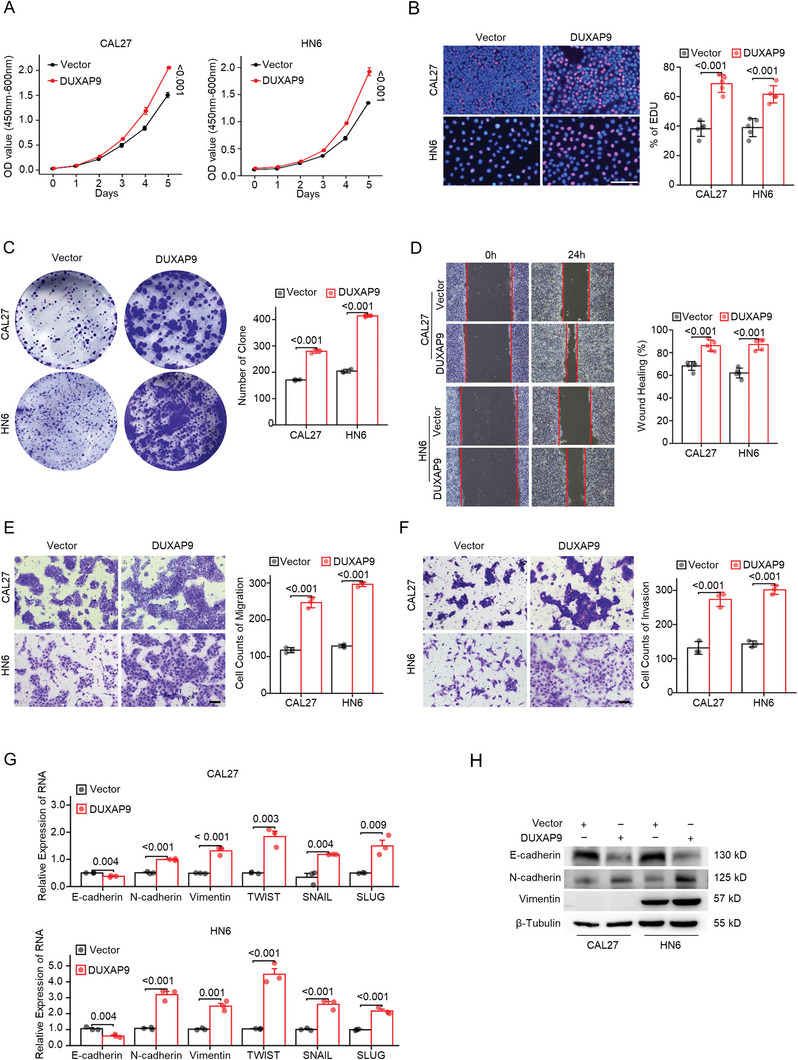
Overexpression of DUXAP9 facilitates OSCC cell growth, migration, and invasion in vitro. A–C) The proliferation of CAL27 and HN6 cells transfected with DUXAP9 overexpression (DUXAP9) or control (Vector) was evaluated by CCK‐8 assays A), EdU assays B), and colony‐formation assays C). Scale bars, 100 µm. D,E). The migration behavior of CAL27 and HN6 cells transfected with DUXAP9 or vector was assessed by wound healing assays D) and transwell assays E). Scale bars, 100 µm. F) The invasion ability of CAL27 and HN6 cells transfected with DUXAP9 or vector was determined by Transwell assays. Scale bars, 100 µm. G,H) The expression of EMT markers as indicated was measured by qRT‐PCR G) and western blot analysis H) in DUXAP9‐ and vector‐expressing OSCC cells. Data are presented as the mean ± SD from three independent experiments. Data in (A–G) were calculated by two‐tailed unpaired Student's *t*‐test.

Knockdown of DUXAP9 suppresses cell proliferation in CAL27 and HN6 cells, as demonstrated by the cell counting kit 8 (CCK8) assay (**Figure** **2**A), EdU incorporation by fluorescence microscopy (Figure 2B) and colony formation assay (Figure 2C). These results suggest that DUXAP9 is required for cell proliferation. The results of wound healing and transwell assays demonstrated that the knockdown of DUXAP9 suppressed cell migration and invasion in CAL27 and HN6 cells (Figure 2D–F). In addition, qRT‐PCR analyses (Figure 2G) and western blotting (Figure 2H) showed that the epithelial‐mesenchymal transition (EMT) markers N‐cadherin, Vimentin, TWIST, SNAIL, and SLUG are decreased with DUXAP9 knockdown, while E‐cadherin is increased. In contrast, we used DUXAP9 lentiviral expression vectors to overexpress DUXAP9 in CAL27 and HN6 cells (Figure S2B, Supporting Information). Overexpression of DUXAP9 enhanced cell proliferation (**Figure** **3**A–C), migration, and invasion (Figure 3D–F) in CAL27 and HN6 cells. Moreover, DUXAP9 overexpression increases N‐cadherin, Vimentin, TWIST, SNAIL, and SLUG expression and decreases E‐cadherin expression (Figure 3G,H).

**Figure 4:**
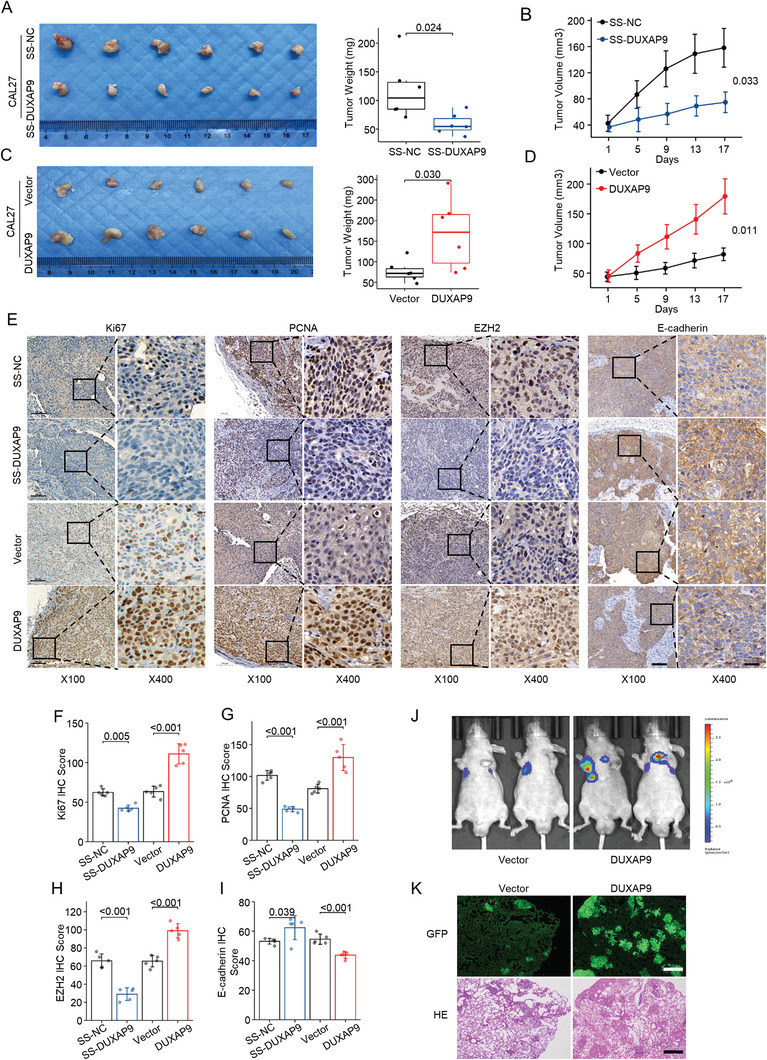
DUXAP9 promoted xenograft tumor growth and metastasis of OSCC cells. A,B) The volumes and weights A) and the growth curves B) of tumors derived from CAL27 cells transfected with SS‐NC or SS‐DUXAP9 ASO were measured, and representative tumor images were taken. *n* = 6/group. C,D) The volumes and weights C) and the growth curves D) of tumors derived from CAL27 cells transfected with vector or DUXAP9 plasmids were measured, and representative tumor images were taken. *n* = 6/group. E) Immunohistochemical staining of Ki67, PCNA, EZH2, and E‐cadherin in tumors derived from CAL27 cells transfected with SS‐NC and SS‐DUXAP9 ASO or control‐ and DUXAP9‐expressing vectors, respectively, Scale bars, 100 µm (left), 25 µm (right). F–I) The expression of Ki67 F), PCNA G), EZH2 H), and E‐cadherin I) was determined by the IHC score in the indicated groups. J) Representative bioluminescence images of lung metastasis in mice injected with CAL27 cells stably expressing vector or DUXAP9 via the tail vein. K) Representative images of H&E staining and GFP fluorescence in the lungs of nude mice injected with CAL27 cells overexpressing vector or DUXAP9. Scale bars, 100 µm. Data in (B) and (D) are presented as the mean ± SEM, and data in (A), (C), and (F–I) are presented as the mean ± SD from three independent experiments. Data in (A–D) and (F–I) were calculated by two‐tailed unpaired Student's *t*‐test.

To test DUXAP9 expression in the regulation of tumorigenesis in vivo, we subcutaneously injected DUXAP9 knockdown OSCC cells into null mice and monitored tumor formation for 3 weeks. The knockdown of DUXAP9 significantly reduced tumor volumes and weights in the knockdown group compared with the control group. In contrast, significantly larger tumor volumes and weights were observed after overexpression of DUXAP9 (**Figure** **4**A–D). In addition, Ki67, PCNA, and EZH2 staining is stronger in DUXAP9‐overexpressing tumors and weaker in DUXAP9‐knockdown tumors, while the opposite expression pattern of E‐cadherin was revealed in these tumors (Figure 4E–I). These results further confirmed the critical role of DUXAP9 in the promotion of tumor formation.

To determine whether DUXAP9 affects OSCC cell metastasis, we established a CAL27 reporter cell line that expressed a GFP‐luciferase fusion protein and further stably overexpressed the control or DUXAP9 gene in this line. The cells were injected intravenously into nude mice. Strikingly, we observed that more DUXAP9‐overexpressing reporter cells than vector control cells metastasized to the lungs of nude mice, as illustrated by bioluminescence imaging (Figure 4J). The presence of metastatic nodules in the mouse lungs was confirmed and counted by GFP fluorescence and H&E staining. The mice injected with DUXAP9‐overexpressing reporter cells form more tumor nodules in their lungs than the mice injected with vector cells (Figure 4K).

### 2.3 DUXAP9 is Transcriptionally Activated by YY1 in OSCC Cells

**Figure 5:**
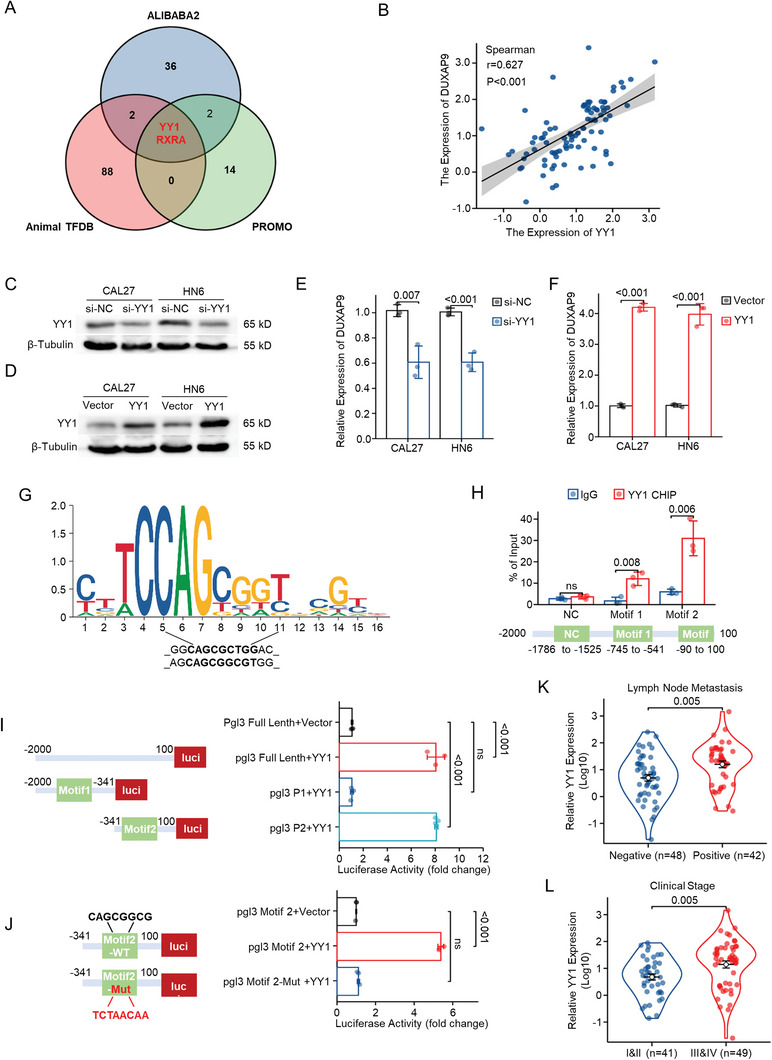
YY1 transcriptionally activates DUXAP9 expression. A) The transcriptional factors that regulate DUXAP9 were predicted using AliBaba2.1, Animal TFDB, and PROMO. B) The correlation between DUXAP9 and YY1 gene expression was analyzed in 90 OSCC patients by qRT‐qPCR. C,D) Western blot analysis of YY1 expression in CAL27 and HN6 cells transfected with control (si‐NC) or siRNA targeting YY1 (si‐YY1) C) or control (vector) or YY1 overexpression (YY1) vector D). E,F) qRT‐qPCR analysis of DUXAP9 in CAL27 and HN6 cells transfected with si‐NC, siYY1 E) or vector, YY1 F), respectively. G) The YY1 binding motif was predicted by JASPAR. H) ChIP–qPCR analysis of the occupation of YY1 in two predicted motifs in the DUXAP9 promoter in CAL27 cells. I) 293T cells were cotransfected with YY1 and luciferase vectors containing DUXAP9 full and truncated promoters. The relative luciferase activity was determined using a dual luciferase reporter system. J) The consensus sequence of YY1 in binding motif 2 of the DUXAP9 promoter was mutated, and the relative luciferase activity from wild‐type and mutant vector‐transfected cells was measured by a dual luciferase reporter system. K,L) The expression of YY1 in OSCC tissues classified according to lymph node metastasis K) and clinical stage L). Data in (B) were calculated by the Spearman correlation test. Data in (E), (F), (H), (I), and (J) were calculated by two‐tailed unpaired Student's *t*‐test. Data in (K,L) were calculated by the Wilcoxon rank‐sum test. Data are presented as the mean ± SD from three independent experiments.

**Figure 6:**
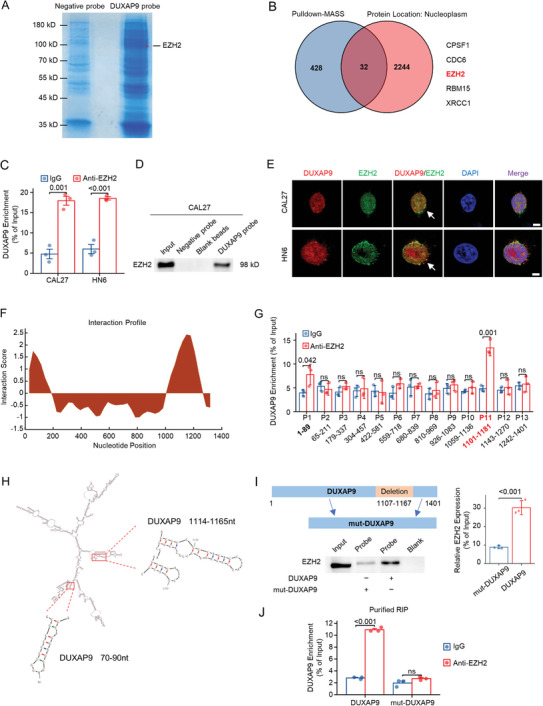
Physical interaction between DUXAP9 and EZH2 in OSCC cells. A) DUXAP9‐interacting proteins were separated by SDS‐PAGE followed by an endogenous DUXAP9 RNA pull‐down assay and manifested by Coomassie Brilliant Blue staining. The distinct protein bands in the gel were excised, dissolved, and subjected to mass spectrometry. The red asterisk denotes the location of EZH2. B) Venn diagram shows a group of nucleoplasm‐located proteins identified by proteomic analysis. The five proteins listed are considered candidates for DUXAP9 binding proteins. C) RIP‐qPCR assay using IgG or anti‐EZH2 antibody shows the enrichment of DUXAP9 expression in the precipitated EZH2 binding complex. D) RNA pull‐down assays with biotin‐labeled DUXAP9 probes show the interaction between DUXAP9 and EZH2 in CAL27 cells. E) Confocal FISH and IF images showing the colocalization of EZH2 (green) and DUXAP9 (red) in CAL27 and HN6 cells. Scale bars, 5 µm. F) Predicted DUXAP9 interaction region using catRAPID. G) qRT‐PCR analysis of DUXAP9 enrichment by RIP assay using anti‐EZH2 in CAL27 cells. Thirteen specific primers for DUXAP9 were used to detect the binding region of DUXAP9. H) Predicted secondary structure of DUXAP9 using Mfold software. I) Western blot followed by RNA pull‐down assay of DUXAP9‐ or mutant DUXAP9 (mut‐DUXAP9)‐transfected cells. The construction of the mutant DUXAP9 vector is shown above, and the expression level of DUXAP9‐associated EZH2 protein is shown below. J) In vitro reconstituted RIP‐qPCR assay using IgG or EZH2 antibody shows the enrichment of DUXAP9 expression in the precipitated EZH2 binding complex. Data are presented as the mean ± SD from three independent experiments. Data in (C), (G), and (J) were calculated by two‐tailed unpaired Student's *t*‐test.

**Figure 7:**
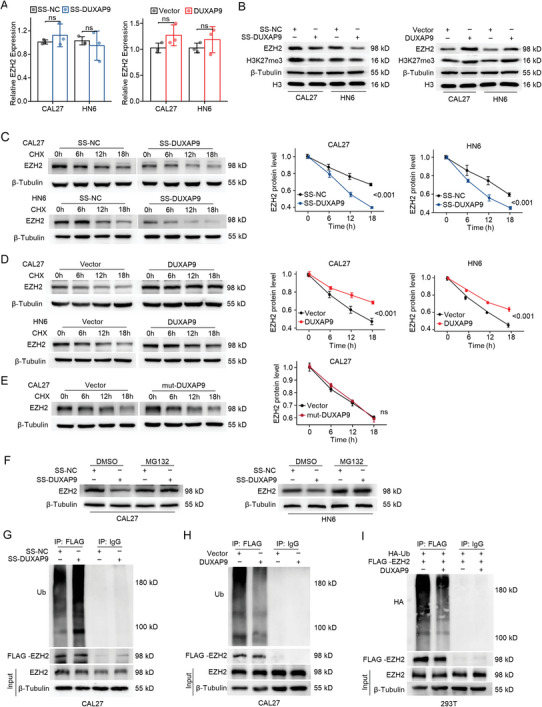
DUXAP9 increases EZH2 protein expression via inhibition of its proteasomal degradation. A) The mRNA expression of EZH2 was measured by qRT‐PCR in CAL27 and HN6 cells transfected with SS‐NC or SS‐DUXAP9 ASO (left) or control‐ or DUXAP9‐overexpressing vectors (right). B) The protein expression of EZH2 was detected by western blot in CAL27 and HN6 cells transfected with SS‐NC or SS‐DUXAP9 ASO (left) or control or DUXAP9 overexpressing vectors (right). C–E) Western blot shows EZH2 protein in CAL27 and HN6 cells transfected with SS‐NC or SS‐DUXAP9 ASO C) or control‐ or wild type DUXAP9‐ D) or mutant DUXAP9‐ E) overexpressing vectors and treated with CHX (20 µg mL^−1^) for the indicated time (left). The quantification of the EZH2 degradation rate was measured by grayscale analysis (right). F) Western blot showing EZH2 protein in CAL27 and HN6 cells transfected with SS‐NC or SS‐DUXAP9 and treated with MG132 (20 µm for 6 h). G, H) Ubiquitination of EZH2 in DUXAP9‐silenced G) and DUXAP9‐overexpressing H) CAL27 cells after MG132 treatment (20 µm for 6 h) was detected by western blot analysis. I) Ubiquitination of EZH2 in 293T cells transfected with EZH2 and DUXAP9 after MG132 treatment (20 µm for 6 h) was detected by western blot analysis. Data are presented as the mean ± SD from three independent experiments. Data in (A) and (C–E) were calculated by two‐tailed unpaired Student's *t*‐test.

**Figure 8:**
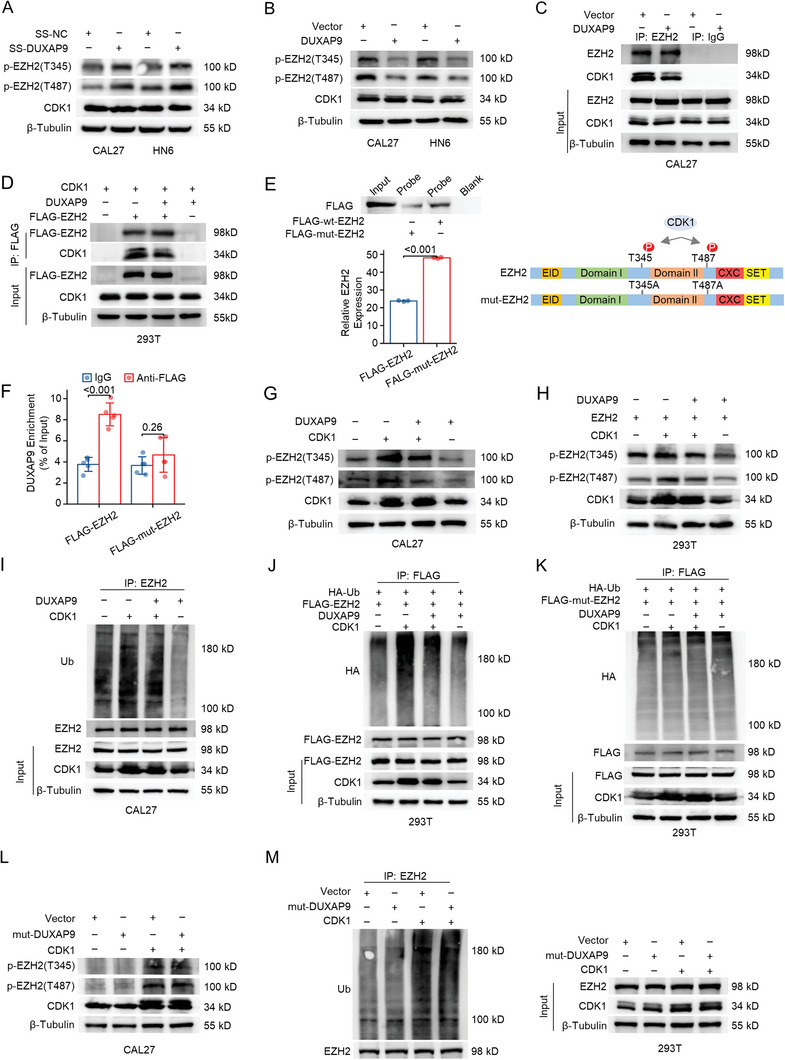
DUXAP9 suppresses EZH2 degradation via inhibition of the phosphorylation (Thr345/Thr487) of EZH2. A,B) Western blot showing the levels of p‐EZH2 (T345), p‐EZH2 (T487), and CDK1 in DUXAP9‐silenced A) and DUXAP9‐overexpressing B) CAL27 and HN6 cells treated with MG132 (20 µm for 6 h). C,D) Western blot showing the interaction between EZH2 and CDK1 in control and DUXAP9‐overexpressing CAL27 C) and 293T D) cells treated with MG132 (20 µm for 6 h) in a coimmunoprecipitation assay using an anti‐EZH2 antibody. E,F) The binding of DUXAP9 to mutant EZH2 or wild‐type EZH2 was detected by RNA pull‐down assay E) and RIP‐qPCR assay F). The diagram shows the mutation site of EZH2. G,H) Western blot showing the levels of p‐EZH2 (T345), p‐EZH2 (T487), and CDK1 in the different combination of transfection of DUXAP9‐ and CDK1 overexpressing vectors in CAL27 G) and 293T H) cells transfected with different combinations of DUXAP9‐ and CDK1‐overexpressing vectors and treated with MG132 (20 µm for 6 h). I,J) Western blot showing the ubiquitination of EZH2 in the different combination of transfection of DUXAP9‐ and CDK1 overexpressing vectors in CAL27 I) and 293T J) cells transfected with different combinations of DUXAP9‐ and CDK1‐overexpressing vectors and treated with MG132 (20 µm for 6 h). K) Western blot shows the ubiquitination of mutant EZH2 in the different combination of transfection of DUXAP9‐ and CDK1 overexpressing vectors in 293T cells transfected with different combinations of DUXAP9‐ and CDK1‐overexpressing vectors and treated with MG132 (20 µm for 6 h). L) Western blot showing the levels of p‐EZH2 (T345), p‐EZH2 (T487), and CDK1 in the different combinations of mutant DUXAP9‐ and CDK1‐overexpressing vectors treated with MG132 (20 µm for 6 h). M) Western blot analysis of the ubiquitination of EZH2 in 293T cells transfected with different combinations of mutant DUXAP9‐ and CDK1‐overexpressing vectors and treated with MG132 (20 µm for 6 h). Data are presented as the mean ± SD from three independent experiments. Data in (F) were calculated by two‐tailed unpaired Student's *t*‐test.

**Figure 9:**
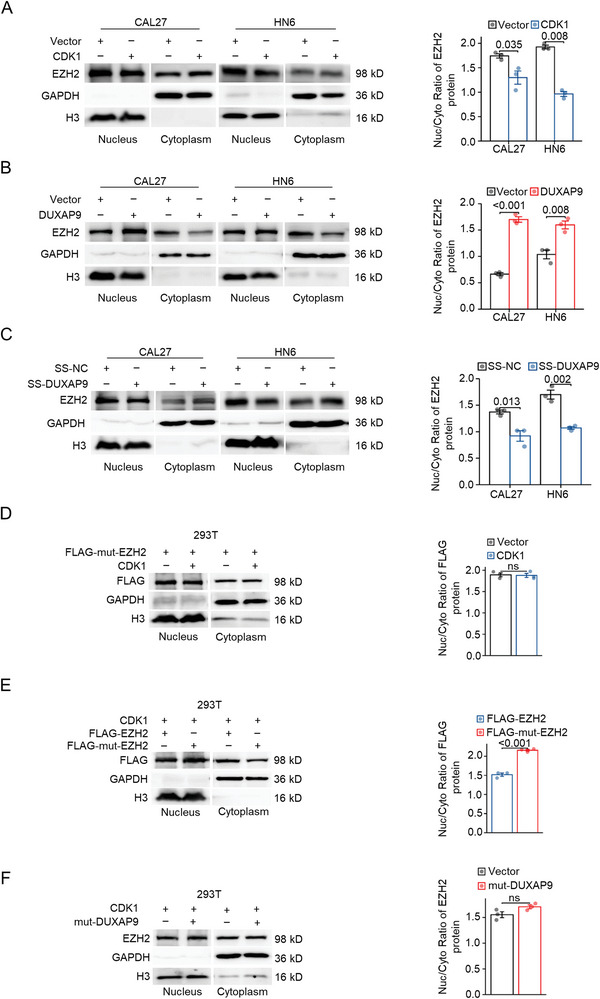
DUXAP9 suppresses EZH2 degradation via nuclear‐to‐cytoplasmic translocation. A–C) Western blot showing the levels of nuclear and cytoplasmic EZH2 in CDK1‐ A) and DUXAP9‐ B) overexpressing and DUXAP9 knockdown C) CAL27 and HN6 cells. Quantification of the EZH2 nucleus/cytoplasm ratio by grayscale analysis is shown on the right. D) Western blot shows the levels of nuclear and cytoplasmic mutant EZH2 in vector‐ or CDK1‐overexpressing 293T cells. E) Western blot shows the levels of nuclear and cytoplasmic wild‐type EZH2 and mutant EZH2 in CDK1‐overexpressing 293T cells. F) Western blot shows the levels of nuclear and cytoplasmic EZH2 in 293T cells transfected with control or mutant DUXAP9‐overexpressing vectors in combination with CDK1‐overexpressing vector. Data are presented as the mean ± SD from three independent experiments. Data in (A–F) were calculated by two‐tailed unpaired Student's *t*‐test.

**Figure 10:**
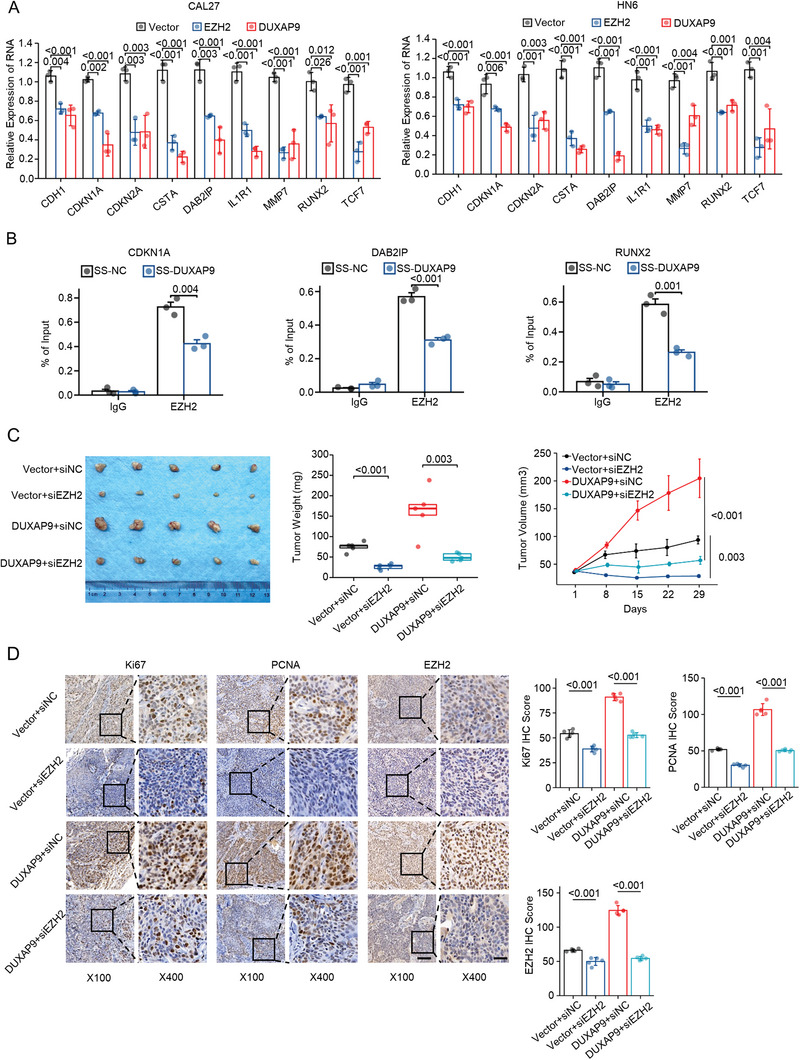
DUXAP9 promotes the proliferation and invasion of OSCC cells by mediating EZH2 expression and function. A) The mRNA expression of 9 known EZH2 target genes was analyzed by qRT‐PCR assays in CAL27 and HN6 cells transfected with vector, DUXAP9, and EZH2. B) ChIP‐qPCR assay of EZH2 or IgG occupancy at the CDKN1A, DAB2IP, and RUNX2 loci in CAL27 cells transfected with SS‐NC or SS‐DUXAP9. C) The volumes, weights and growth curves of tumors derived from CAL27 cells transfected with control‐ or DUXAP9‐expressing vectors and siRNAs targeting NC or EZH2 were measured and imaged; D,E) The expression of Ki67, PCNA, and EZH2 was determined by the IHC score in the indicated groups. *n* = 5/group, Scale bars, 100 µm (left), 25 µm (right). Data in (C) are presented as the mean ± SEM, and data in (A,B) and (D) are presented as the mean ± SD from three independent experiments. Data were calculated by two‐tailed unpaired Student's *t*‐test.

**Figure 11:**
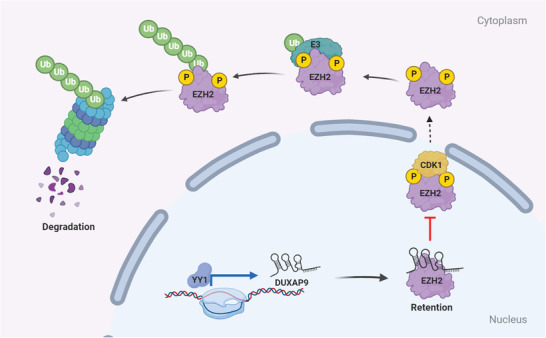
Schematic depicting YY1‐induced DUXAP9 drives OSCC by blocking CDK1‐mediated EZH2 degradation. DUXAP9 orchestrates a different biological function of CDK1‐mediated phosphorylation of the T345 and T487 sites of EZH2 in controlling the protein stability of EZH2 via nuclear to cytoplasmic translocation, suggesting the importance of lncRNA regulation at the posttranslational level in OSCC progression.

It is generally appreciated that the activation of oncogenic transcription factors (TFs) contributes to the aberrant expression of lncRNAs in cancer, and hence, we searched for transcription factors that potentially regulate the upregulation of DUXAP9 in OSCC. We predicted TFs occupying the DUXAP9 promoter region by using AliBaba2.1 (http://gene‐regulation.com), ALGEEN‐PROMO (http://alggen.lsi.upc.es/cgi‐bin/promo_v3/promo/promoinit.cgi?dirDB = TF_8.3), and AnimalTFDB3 (http://bioinfo.life.hust.edu.cn/AnimalTFDB#!/species). The results revealed that two TFs (RXRA and YY1) are predicted to bind to the promoter of DUXAP9 (**Figure** 1, 2, 3, 4, 5, 6, 7, 8, 9, 10, 11). Then, we analyzed the expressional correlation between RXRA, YY1, and DUXAP9 using the SJTU cohort and TCGA cohort and found that only YY1 has a significant positive expressional correlation with DUXAP9 (Figure 5B; and Figure S2C,D, Supporting Information).

Knockdown of YY1 in OSCC cells suppresses the expression of DUXAP9. Conversely, YY1 overexpression upregulates DUXAP9 expression in OSCC cells (Figure 5C–F). JASPAR 2020 prediction further showed that there are two binding motifs for YY1 within −2000 to 100 bp of the promoter region of DUXAP9 (Figure 5G). To verify the binding sites of YY1 to the DUXAP9 promoter, we performed chromatin immunoprecipitation (ChIP) and luciferase assays. Primers targeting the promoter region of DUXAP9, which contains the predicted YY1 binding sites, were designed. ChIP assay results indicated that YY1 was highly enriched in the −90 to 100 bp region of the DUXAP9 promoter region (Figure 5H). To further clarify the YY1 transcriptional binding sites in the promoter region of DUXAP9, we constructed a series of promoter truncated promoter luciferase reporter vectors. The pgl3‐P2 luciferase reporter with an adjacent YY1 binding motif was significantly activated under YY1 expression, while the pgl3‐P1 luciferase reporter with a distant motif was not activated in OSCC cells. To verify that the consensus sequence of the YY1 binding motif is required for mediating DUXAP9 expression by YY1, a mutated luciferase reporter of the predicted YY1 binding site was constructed. The mutant constructs were transfected into OSCC cells, and the mutation of Motif2 from 8 to 23 bp of the DUXAP9 promoter showed significantly lower luciferase activity than the WT control (Figure 5I,J). Furthermore, we examined the clinical relevance of YY1 in OSCCs. YY1 expression in OSCC tissues is higher than that in adjacent normal tissues (Figure S2E, Supporting Information). We revealed that YY1 expression is also significantly higher in paired OSCC samples from the SJTU cohort with lymph node metastasis (Figure 5K) and advanced clinical stage (Figure 5L) but not in moderate/poor pathological differentiation samples (Figure S2F, Supporting Information). Together, these results reveal that YY1 transcriptionally regulates DUXAP9 expression in OSCC cells.

### 2.4 Physical Interaction Between DUXAP9 and EZH2

To explore the mechanisms by which DUXAP9 regulates OSCC tumorigenesis, RNA pull‐down experiments were performed using a mixture of 6 antisense probes (Table S3, Supporting Information) against endogenous DUXAP9 in CAL27 cells, and the precipitated RNA‐protein complex was identified via mass spectrometry analysis (Figure **6**A). A total of 428 proteins were identified, and 32 proteins are localized in the nucleoplasm and considered candidates for DUXAP9 binding partners. By reviewing previous studies, we found that CPSF1,^[^ ^18^ ^]^ CDC6,^[^ ^19^ ^]^ EZH2,^[^ ^5b^ ^]^ RBM15,^[^ ^20^ ^]^ and XRCC1^[^ ^21^ ^]^ were reported to be highly expressed in HNSCC and correlated with the malignant progression of HNSCC (Figure 6B). Our previous targeted drug screening identified EZH2 as a critical regulator in OSCC progression.^[^ ^5a^ ^]^ The RIP‐qPCR assay using anti‐EZH2 antibody or IgG control in CAL27 and HN6 cells (Figure 6C) and RNA pull‐down assays with biotin‐labeled DUXAP9 probes in CAL27 cell lysates (Figure 6D) confirmed that DUXAP9 physically binds to EZH2. Confocal microscopy also showed the colocalization of DUXAP9 with EZH2 in the nucleus of OSCC cells (Figure 6E). Furthermore, the potential binding sites between DUXAP9 and EZH2 were predicted by using an algorithm to estimate the binding propensity of protein‐RNA pairs (catRAPID, https://service.tartaglialab.com), showing that DUXAP9 had a high protein–RNA interaction propensity (Figure 6F). According to the predicted DUXAP9 binding site, a set of primers was designed for the RIP‐qPCR experiment. Among the 13 pairs of primers, only primer pairs 1 and 13 designed for the 1–89 nt and 1101–1181 nt regions of DUXAP9 successfully amplified the DUXAP9 segment (Figure 6G). Based on these results, we speculate that the nucleotides at base pairs 1–89 nt and 1101–1181 nt of DUXAP9 might be responsible for binding with EZH2. Using Mfold, an RNA secondary structure prediction tool (http://unafold.rna.albany.edu/?q = mfold), we found that nucleotides 70–90 nt and 1114–1165 nt of DUXAP9 may form a stem‐loop structure, which could serve as an RNA‐protein binding site (Figure 6H). Furthermore, we found that DUXAP9 with a 1107–1167 nt deletion (mut‐DUXAP9) largely attenuates the binding efficiency to EZH2 by RNA pull‐down assay (Figure 6I). The in vitro reconstituted RIP assay using recombinant EZH2 protein and in vitro transcribed wild‐type and mutant DUXAP9 RNA further verified the direct interaction between DUXAP9 and EZH2 (Figure 6J), suggesting that the nucleotides at base pairs 1114–1165 nt of DUXAP9 may be the major binding site of EZH2.

### 2.5 DUXAP9 Increases EZH2 Protein Stability Via Inhibition of Its Proteasomal‐Mediated Degradation

To study whether DUXAP9 regulates the expression level of EZH2, the transcriptional and translational levels of EZH2 in OSCC cells were detected by RT‐qPCR and Western blotting assays after silencing or overexpressing DUXAP9, respectively. Notably, we found that DUXAP9 positively regulates EZH2 protein levels but not the mRNA level of EZH2, indicating that DUXAP9 may post‐translationally regulate EZH2 expression in OSCC cells (Figure **7**A,B).

LncRNAs have previously been proven to regulate the protein stability of their binding partners.^[^ ^22^ ^]^ To further elucidate the mechanism by which DUXAP9 upregulates EZH2 expression post‐translationally, the stability of the EZH2 protein in CAL27 and HN6 cells was examined upon cycloheximide (CHX) treatment, which inhibits the synthesis of de novo proteins. After CHX treatment, EZH2 protein degrades more rapidly in SS‐DUXAP9 OSCC cells with a shortened half‐life (Figure 7C), while overexpression of full‐length DUXAP9, but not mut‐DUXAP9, in OSCC cells significantly increased EZH2 protein stability and extended the half‐life, indicating that DUXAP9 expression promotes EZH2 stability (Figure 7D,E). Most of the protein is degraded through the proteasome pathway. Using the proteasome inhibitor MG132, we found that the decreased EZH2 expression induced by SS‐DUXAP9 transfection was completely rescued upon MG132 treatment in CAL27 and HN6 cells (Figure 7F). Furthermore, MG132 increased EZH2 expression in control cells but not in DUXAP9‐overexpressing cells (Figure S3A, Supporting Information). These results suggest that DUXAP9 increases EZH2 protein expression by inhibiting its proteasomal degradation.

Ubiquitination of proteins is required for their proteasomal degradation and plays an important role in the progression of cancer.^[^ ^23^ ^]^ Immunoprecipitation of EZH2 followed by antiubiquitin immunoblotting demonstrated that ubiquitinated EZH2 was markedly increased in SS‐DUXAP9‐transfected cells, whereas EZH2 ubiquitination was significantly decreased in DUXAP9‐overexpressing cells (Figure 7G–I). Therefore, we concluded that DUXAP9 inhibits the ubiquitination and proteasomal degradation of EZH2 and leads to the upregulation of EZH2 expression in OSCC cells.

### 2.6 DUXAP9 suppresses EZH2 degradation via inhibition of EZH2 phosphorylation and nuclear‐to‐cytoplasmic translocation

It has been reported that the phosphorylation of EZH2 (p‐EZH2) at Thr345 and Thr487 mediated by CDK1 is required for ubiquitination and subsequent degradation by the ubiquitin‒proteasome pathway.^[^ ^24^ ^]^ We confirmed that phosphorylated EZH2 (Thr345/487) and ubiquitinated EZH2 were elevated in CDK1‐overexpressing cells (Figure S3B–S3E, Supporting Information). These studies led us to hypothesize that the DUXAP9–EZH2 interaction may inhibit EZH2 ubiquitination and degradation by suppressing EZH2 Thr345 and Thr487 phosphorylation.

To address this hypothesis, we performed DUXAP9 knockdown and overexpression assays and found that DUXAP9 knockdown markedly increased Thr345/487‐phosphorylated EZH2, while overexpression of DUXAP9 decreased Thr345/487‐phosphorylated EZH2 in CAL27 and HN6 cells. Meanwhile, there was no difference in CDK1 expression between either SS‐DUXAP9‐transfected cells or DUXAP9‐overexpressing cells and control cells (**Figure** **8**A,B). Although our results show that DUXAP9 does not directly interact with CDK1 (Figure S3F, Supporting Information), overexpression of DUXAP9 remarkably suppresses the interaction between EZH2 and CDK1 (Figure 8C,D). The EZH2 mutant at threonine 345/487 (T345A/T487A) (mut‐EZH2) decreases the binding of EZH2 and DUXAP9, implying that DUXAP9 may impede CDK1 binding and phosphorylating EZH2 (Figure 8E,F). When CDK1 was transfected and overexpressed in CAL27 and 293T cells, the reduced levels of phosphorylated EZH2 (Thr345/487) (Figure 8G,H) and ubiquitinated EZH2 (Figure 8I,J) reduced by DUXAP9 overexpression are largely reversed. These findings suggest that DUXAP9‐EZH2 binding is probably able to attenuate the EZH2 interaction with CDK1, inhibit the phosphorylation of EZH2 at Thr345 and Thr487, and ultimately inhibit the ubiquitination and degradation of EZH2 through the ubiquitin‐proteasome pathway.

To further confirm this hypothesis, we compared the effect of DUXAP9 overexpression on CDK1‐induced EZH2 ubiquitination levels in cells expressing wild‐type EZH2 (wt‐EZH2) or phosphorylation site mutant EZH2 (T345A/T487A) (mut‐EZH2). Importantly, neither DUXAP9 nor CDK1 alters the phosphorylation and ubiquitin enrichment of mut‐EZH2 (Figure 8K). We also observed that mut‐DUXAP9 expression in cells could not alter the phosphorylation and ubiquitination of EZH2 (Figure 8L,M). In total, these results indicate that the 1114–1165 nt region of DUXAP9 mainly interacts with EZH2 at the Thr345/487 phosphorylation site, blocking its phosphorylation and subsequent ubiquitination induced by CDK1.

Our results suggested that CDK1‐mediated Thr345/487 phosphorylation may lead to its nuclear‐to‐cytoplasmic translocation for degradation. Indeed, the separation of nuclear and cytoplasmic fractionation in OSCC cells showed more cytoplasmic EZH2 in CDK1‐overexpressing cells than in control cells (**Figure** **9**A). Furthermore, overexpressing DUXAP9 in OSCC cells significantly reduces EZH2 expression in the cytoplasm and increases EZH2 expression in the nucleus (Figure 9B). Consistently, SS‐DUXAP9 in CAL27 and HN6 cells significantly decreases EZH2 in the nucleus and increases EZH2 in the cytoplasm (Figure 9C). This nuclear‐to‐cytoplasmic translocation of EZH2 may be influenced by its phosphorylation and may be responsible for its ubiquitination and degradation. After transfection of mut‐EZH2 into 293T cells, this CDK1‐induced nuclear‐to‐cytoplasmic translocation of EZH2 is significantly abolished (Figure 9D,E). A similar result was observed after transfection of mut‐DUXAP9 (Figure 9F).

### 2.7 DUXAP9 Promotes the Proliferation and Invasion of OSCC Cells in an EZH2‐Dependent Manner

To determine whether DUXAP9 promotes OSCC development by mediating EZH2 expression and EZH2‐associated PRC2 complex activity. First, the mRNA expression of 9‐well‐known EZH2 target genes was analyzed by qRT‐PCR assays in vector‐, DUXAP9‐ and EZH2‐overexpressing CAL27 and HN6 cells (**Figure** **10**A). The results showed that the expression of these target genes is decreased in both EZH2‐overexpressing and DUXAP9‐overexpressing cells, suggesting that DUXAP9 functions through EZH2 expression. It is possible that DUXAP9 regulates EZH2 target genes by modulating PRC2 complex activity. To validate this hypothesis, we performed EZH2 ChIP‐qPCR assays in DUXAP9 knockdown and control CAL27 cells (Figure 10B). We found that silencing DUXAP9 reduced EZH2 binding to its target genes, which indicates that PRC2 complex function is compromised in DUXAP9 knockdown cells.

Knockdown of DUXAP9 using SS‐DUXAP9 in CAL27 and HN6 cells dramatically suppressed the ability of EZH2 to promote OSCC cell proliferation and migration, and silencing of EZH2 using siEZH2 in CAL27 and HN6 cells also remarkably blocked the effect of DUXAP9 on promoting cell proliferation (Figure S4A,B, Supporting Information), colony formation (Figure S4C, S4D, Supporting Information), migration (Figures S4E,F and S5A,B, Supporting Information), and invasion (Figure S5C,D). Silence of EZH2 significantly reduced the volumes and weights of tumors formed from CAL27 cells overexpressing DUXAP9 (Figure 10C) and decreased the expression levels of the Ki67, PCNA, and EZH2 proteins (Figure 10D). These results implied that DUXAP9 induces OSCC cell proliferation, migration, and invasion in an EZH2‐dependent manner.

Collectively, our results suggest that DUXAP9 binds and inhibits the phosphorylation (Thr345/487) and ubiquitination of EZH2, thereby suppressing its nuclear‐to‐cytoplasm translocation for proteasomal degradation (**Figure** **11**).

## 3 Discussion

LncRNA has been reported to be notably involved in OSCC initiation and development by non‐negligible regulatory element effects.^[^ ^25^ ^]^ Specifically, lncRNAs act as oncogenes or suppressors in OSCC progression due to their complex functions. MALAT1 promotes cellular proliferation, invasion, and metastasis in head and neck squamous cell carcinoma by TGF‐*β*‐induced STAT3 activation.^[^ ^26^ ^]^ In addition, MALAT1 inhibited VHL by activating the EZH2/STAT3/Akt axis and then promoted the stabilization and activation of *β*‐catenin and NF‐*κ*B.^[^ ^27^ ^]^ LincRNA‐p21 acts as a tumor suppressor by inhibiting JAK2/STAT3 signaling pathways in head and neck squamous cell carcinoma.^[^ ^28^ ^]^ lnc‐POP1‐1 bound to the minichromosome maintenance deficient 5 (MCM5) protein directly and decelerated MCM5 degradation by inhibiting ubiquitination of the MCM5 protein, which facilitated the repair of DNA damage caused by cisplatin in HNSCC cells.^[^ ^29^ ^]^ DUXAP9, also termed LNMAT1 or LINC01296, has been explored as an oncogene promoting tumor progression in bladder cancer,^[^ ^30^ ^]^ malignant melanoma,^[^ ^31^ ^]^ HNSCC,^[^ ^32^ ^]^ and renal cell carcinoma.^[^ ^14^ ^]^ Recent phenotypic analyses showed that dysregulation of DUXAP9/LINC01296 accelerates the development of HNSCC and predicts a poor prognosis.^[^ ^32^ ^]^ However, whether DUXAP9 regulates OSCC progression and the underlying mechanism remain unclear. Here, upregulated and prognosis‐related DUXAP9 was identified in OSCC tissues by a lncRNA transcriptome array and bioinformatics analysis (Figure 1). Furthermore, our results demonstrated that DUXAP9 facilitates proliferation, migration, invasion, and metastasis in both OSCC cell lines and animal models (Figures 2, 3, 4).

YY1 is a zinc‐finger protein that belongs to the GLI‐Krüppel family. It was first identified from a sequence originally isolated as a repressor of the P5 promoter of the adeno‐associated virus.^[^ ^33^ ^]^ Depending on the context, YY1 can function as a transcriptional activator or as an inhibitor in tumorigenesis.^[^ ^34^ ^]^ In OSCC, YY1 expression is associated with poor clinicopathological features. In our study, YY1 expression was found to be positively associated with the expression of DUXAP9, and YY1 was largely recruited to motif 1 (−745 to −541 bp) and motif 2 (−90 to 100 bp) of the DUXAP9 promoter by ChIP assay (Figure 5G,H). However, only motif 2 of the DUXAP9 promoter binding to YY1 activates the transcriptional activity of DUXAP9 (Figure 5I,J). Our findings showed that YY1 is the main factor contributing to the dysregulation of DUXAP9 in OSCC.

In our current study, DUXAP9 was found to bind to EZH2 by pull‐down‐MASS and bioinformatics analysis (Figure 6A,B). Furthermore, the direct and physical interaction of DUXAP9 and EZH2 was revealed by RIP and pulldown assays (Figure 6C,D). Additionally, the colocalization between DUXAP9 and EZH2 in the nucleus was detected in OSCC cell lines (Figure 6E). Then, a weak binding region at 1–69 nt and a strong binding region at 1107–1167 nt of the DUXAP9 transcript were identified. The following mutational assays showed that the 1107–1167 nt region of DUXAP9 contributes to the majority of DUXAP9 activity in interacting with EZH2.

Upregulation of EZH2 results in cancer progression and poor prognosis for patients with tumors.^[^ ^35^ ^]^ Post‐translational modifications (PTMs) are important biological processes in cancer development. Recent evidence indicates that the PTMs of EZH2 are crucial for its protein stability, enzymatic activity, and function in cancer development.^[^ ^36^ ^]^ For example, it was reported that the phosphorylation of T350 of EZH2 in humans catalyzed by CDK1 or CDK2 is necessary for EZH2‐mediated H3K27me3 modifications in prostate cancer cells.^[^ ^37^ ^]^ CDK1‐mediated EZH2 phosphorylation at pT345 and pT487 is not essential for H3K27me3 formation.^[^ ^24^ ^]^ However, pT345 and pT487 of EZH2 were found to accelerate EZH2 ubiquitination by the proteasome pathway in cervical cancer cells.^[^ ^24^ ^]^ In contrast, pT345 and pT487 of EZH2 were found to attenuate oncogenesis and metastasis in breast cancer by reducing EZH2 stability. LncRNA ANCR mediates the instability of EZH2 by enhancing the level of phosphorylation at both Thr‐345 and Thr‐487 in breast cancer cells, with concomitant inhibition of breast cancer cell motility.^[^ ^38^ ^]^ However, the details of how ANCR physically interacts with EZH2 remain unclear. The conformational change of EZH2 due to its interaction with ANCR is speculated to be a potential mechanism that facilitates CDK1‐mediated EZH2 phosphorylation. Our findings demonstrated that DUXAP9 interacts with EZH2 by a 3′‐stem‐loop structure to conceal the phosphorylation site of EZH2 in OSCC cells. Furthermore, the interaction between DUXAP9 and EZH2 enhances the stability of the EZH2 protein (Figure 7A,B). How ANCR and DUXAP9 coordinately regulate EZH2 phosphorylation by CDK1 in OSCC cells needs to be further investigated. After CHX treatment, DUXAP9, but not mut‐DUXAP9, markedly decreases EZH2 degradation in OSCC cells (Figure 7C–E). MG132 treatment rescued the proteasomal degradation of EZH2 after silencing of DUXAP9, suggesting that DUXAP9 inhibits EZH2 ubiquitination and proteasome‐mediated degradation in OSCC cells (Figure 7F). Next, endogenous and exogenous ubiquitination experiments revealed that DUXAP9 suppresses the interaction of CDK1 and EZH2 and CDK1‐mediated phosphorylation of EZH2 at pT345 and pT487 (Figure 8A–D). Notably, the mutation of these phosphorylation sites of EZH2 abolishes the interaction of DUXAP9 and EZH2, proving that DUXAP9 interacts with EZH2 to prevent its phosphorylation by CDK1 (Figure 8K–M). pT345 and pT487 of EZH2 facilitate EZH2 ubiquitination and proteasomal‐mediated degradation. Here, we demonstrated that pT345 and pT487 of EZH2 mediate the nucleus‐to‐cytoplasm translocation of EZH2, which is required for the proteasome‐mediated degradation of EZH2, thus affecting PRC2 complex activity and OSCC pathogenesis (Figures 9 and 10). Together, the above results revealed that DUXAP9 orchestrates a different biological function of CDK1‐mediated phosphorylation of the T345 and T487 sites of EZH2 in controlling the protein stability of EZH2 via nuclear to cytoplasmic translocation, suggesting the importance of lncRNA regulation at the posttranslational level in OSCC progression (Figure 11).

## 4 Conclusions

In conclusion, our study identified a novel nuclear‐localized lncRNA, DUXAP9, that is highly expressed in OSCC. Gain‐ and loss‐of‐function assays demonstrated that DUXAP9 promotes OSCC cell proliferation, invasion and metastasis in vitro and in vivo. Mechanistically, DUXAP9 is transcriptionally activated by YY1 and inhibits CDK1‐mediated phosphorylation of EZH2 at T345 and T487 by directly binding to EZH2. Furthermore, DUXAP9 abolishes the translocation of EZH2 from the nucleus to the cytoplasm, where it undergoes ubiquitination and proteasomal degradation. Overall, our study revealed a novel mechanism by which DUXAP induces EZH2 expression at the posttranslational level, which drives OSCC formation and progression, suggesting that DUXAP9 may be a potential target for OSCC therapy.

## 5 Experimental Section

### Cell Lines and Cell Culture

Five human OSCC cell lines (CAL27, WSU‐HN6, WSU‐HN30, and SCC‐9) and 293T cells were used in this study. WSU‐HN6 (HN6) and WSU‐HN30 (HN30) cells were kindly provided by the University of Maryland Dental School, USA. The CAL27, SCC9, and 293T cell lines were obtained from the ATCC (American Type Culture Collection). CAL27, HN6, HN30, and 293T cells were cultured in Dulbecco's modified Eagle's medium (DMEM; BasalMedia, CN). SCC‐9 cells were cultured in DMEM/F12 (1:1) medium (BasalMedia). The media were supplemented with 10% heat‐inactivated fetal bovine serum (FBS) (BasalMedia), penicillin (100 units mL^−1^), and streptomycin (100 µg mL^−1^). The cells were cultured at 37 °C in a humidified 5% CO_2_ atmosphere.

### Patients and Specimens

The study was approved by the Ethics Committee of Shanghai Ninth People's Hospital affiliated with Shanghai Jiao Tong University, School of Medicine. OSCC tissues and their paired adjacent normal tissues were obtained from the Shanghai Sharing Platform for the Tissue and Bioinformatics Database of Oral Maxillofacial Tumors (http://mdl.shsmu.edu.cn/OMNDB/page/home/home_en.jsp), which was established by the Ninth People's Hospital, Shanghai Jiao Tong University School of Medicine, and the Shanghai Institute of Stomatology (Shanghai, China) (2018‐86‐T77). All tissue samples used for the Sharing Platform were collected from the Department of Oral and Maxillofacial‐Head and Neck Oncology, Ninth People's Hospital, Shanghai Jiao Tong University School of Medicine.

### Affymetrix OE lncRNA Microarray Analysis

The Affymetrix OE lncRNA microarray was performed on an Affymetrix gene chip command console (version 4.0, Affymetrix) software by OEbiotech (Shanghai, P. R. China). In brief, total RNA was quantified by a NanoDrop ND‐2000 (Thermo Scientific), and the RNA integrity was assessed using an Agilent Bioanalyzer 2100 (Agilent Technologies). Sample labeling, microarray hybridization, and washing were performed according to the manufacturer's instructions. In brief, total RNA was transcribed into double‐stranded cDNA, and then cRNAs were synthesized. Then, second‐cycle cDNAs were synthesized from cRNAs. Following fragmentation and biotin labeling, the second‐cycle cDNAs were hybridized onto the microarray. After washing and staining, the arrays were scanned by the Affymetrix Scanner 3000 (Affymetrix). Differentially expressed genes and lncRNAs were then identified through fold change. The threshold set for up‐ and downregulated genes was a fold change ≥2.0.

### Smart Silencer/siRNA or Plasmid Transfection

The Smart Silencer used in the study was designed and synthesized by Guangzhou RiboBio Co., Ltd. (Guangzhou, China) and are listed in Table S2 (Supporting Information). The plasmid, siRNA, and negative control sequences were designed and synthesized by Genomeditech (Shanghai, China). Transfection was performed using Lipofectamine 3000 reagent (Invitrogen) following the manufacturer's instructions.

### RNA Extraction and qRT‐PCR

Total RNA from tissues and cells was extracted using TRIzol reagent (Invitrogen, USA) and used to generate cDNA with HiScript II Q RT SuperMix (Vazyme, CN). Real‐time PCR was performed using a LightCycler 480 Real‐Time PCR System (Roche, USA) with 2x SYBR Green qPCR Master Mix (Bimake, USA) according to the manufacturer's instructions. The RNA levels were normalized against 18S levels. The PCR primers were designed and synthesized by Sangon Biotech (Shanghai) Co., Ltd. and are listed in Table S4 (Supporting Information).

### Lentiviral Transduction and Screening of Stable Strains

DUXAP9 lentiviral expression vectors were constructed by Genomeditech (China). The DUXAP9 lentiviral expression vector, expressing the full length of 1401 bp of DUXAP9, conferred puromycin resistance. Lentiviral transduction was performed following the manufacturer's instructions. After 72 h of transfection, the culture medium was mixed with puromycin at a final concentration of 2–3 µg mL^−1^. After being cultured with puromycin and passaged 2–3 times, the stably stained cells were screened.

### Cell Counting Kit‐8 (CCK‐8) Analysis

Cells transfected for 24 h with Smart Silencer/siRNA or stably lentivirus‐transduced cells were seeded into 96‐well plates at a density of 1000 cells per well in triplicate. The cells were harvested, and 10 µL of CCK‐8 reagent (Dojindo, Kumamoto, Japan) was added to 100 µL of culture medium. The cells were subsequently incubated for 2 h at 37 °C, and the optical density was measured at 450 and 600 nm using a microplate reader (Multiskan Sky Spectrophotometer, Thermo Scientific, USA).

### Colony‐Forming Assay

Cells transfected for 24 h with Smart Silencer/siRNA or stably lentivirus‐transduced cells were seeded into 6‐well plates at a density of 1000 cells per well and incubated for 10–14 days to form cell colonies. The colonies were fixed with 4% paraformaldehyde (Wuhan Servicebio Technology Co., Ltd., China) and stained with 1% crystal violet (Biosharp, China), and those with more than 50 cells were counted under a dissecting microscope.

### 5‐Ethynyl‐2′‐deoxyuridine (EdU) Assay

The treated HN6 and Cal27 cells were incubated in 48‐well plates with 300 µL of 50 µm EdU (RiboBio, Guangzhou, China) per well for 2 h. After fixation with 4% paraformaldehyde and washing, the cells were treated with 300 µL of 1× Apollo reaction for 30 min. Then, the DNA of the cells was stained with DAPI for 5 min and visualized under a fluorescence microscope.

### Wound‐Healing Assay

CAL27 and HN6 cells transfected for 24 h with Smart Silencer/siRNA or lentivirus‐transduced stable cells were plated in 6‐well plates and cultured to confluency. Then, tumor cells were scraped with a P200 tip (0 h), washed with PBS, and cultured with serum‐free DMEM. Five nonoverlapping field images were obtained at 24 h.

### Transwell Migration and Invasion Assays

Cell migration and invasion assays were performed using 24‐well Transwell chambers with 8 µm porosity polycarbonate filters and Transwell insert chambers (Corning, USA) coated or not coated with Matrigel (Corning, USA). A total of 200 µL of cell suspension in serum‐free medium was added into each upper chamber, while 600 µL of DMEM supplemented with 10% FBS was added to the lower chambers as a chemoattractant. After incubating for 24–36 h, the migrated or invaded cells were fixed with 4% paraformaldehyde for 15 min and stained with 1% crystal violet for 30 min. After the cells on the upper surface of the filter were removed, at least five randomly selected microscopic fields of fixed cells per filter were imaged using an inverted phase‐contrast microscope. The cells were counted, and the average was calculated.

### RNAScope

An RNAScope assay was performed to detect single‐molecule RNA using the RNAScope Assay Kit (Advanced Cell Diagnostics, CA). Sixteen paired double‐Z oligonucleotide probes targeting 141–1381 nt of DUXAP9 were designed by ACD. The experiment was performed according to the manufacturer's instructions.

### RNA FISH and In Situ Hybridization

Fluorescence‐labeled probes for DUXAP9, 18S rRNA, and U6 RNA were designed and synthesized by Guangzhou RiboBio Co., Ltd, and FISH experiments were performed using a Ribo Fluorescent In Situ Hybridization kit (R11060.6, RiboBio). For the colocalization assay, anti‐EZH2 antibody (#5246, CST, dilution 1:50) was added to the reaction, followed by Fluor secondary antibodies (AS011, Abclonal, dilution 1:200) and DAPI. Images were acquired on an SP8 laser‐scanning confocal microscope (Leica Microsystems, Germany).

### Isolation of Nuclear and Cytoplasmic RNA

Nuclear, cytoplasmic and total RNA was isolated using a PARIS kit (Thermo Fisher Scientific, USA) following the manufacturer's instructions. After purification and DNase I treatment, RNA from the isolated nuclear and cytoplasmic fractions was reverse transcribed and used for PCR as described above. MALAT1, NEAT1, and U6 were used as endogenous controls for the nucleus, while *β*‐actin and GAPDH were used as endogenous controls for the cytoplasm. The primers used for PCR are listed in Table S4 (Supporting Information).

### RNA Immunoprecipitation Analysis (RIP)

RIP was performed according to the manufacturer's instructions of the Magna RIP RNA‐Binding Protein Immunoprecipitation Kit (Millipore, USA). After cell lysis with RIP lysis buffer, 100 µL of the lysate was incubated with RIP buffer containing magnetic beads, which were conjugated with human anti‐EZH2 antibody (#5246, CST, USA) and normal rabbit IgG (Millipore). Among the antibodies, IgG was considered a negative control. The samples were then incubated with proteinase K to isolate immunoprecipitated RNA. Finally, the target RNA was extracted and purified for further study by qRT‐PCR assays. An in vitro reconstituted RIP assay was conducted with human recombinant EZH2 protein (#TP302054, Origene, US) and DUXAP9 transcribed by a T7 High Yield RNA Transcription Kit (Beyotime, China). The primers used for PCR are listed in Table S4 (Supporting Information).

### UV Crosslinking‐Immunoprecipitation Assay (CLIP)

CAL27 cells were cultured in medium with 4‐thiouridine (100 µm) for 16 h. Then, the cells were washed twice with cold PBS, crosslinked with UA (365 nm, 150 mJ cm^−2^) and lysed with NP‐40 lysis buffer containing protease inhibitors and 1 mm dithiothreitol. RNase T1 was added to the supernatant at a final concentration of 1 U µL^−1^ and incubated at 22 °C for 5 min. Then, EZH2 antibody was added and incubated at 4 °C with rotation for 4 h. Protein A/G magnetic beads were incubated with 10 µg of EZH2 antibody or normal rabbit IgG in NT2 buffer at 4 °C. RNase T1‐treated cell lysate was incubated with the washed antibody–bead complex at 4 °C for 4 h, and then the beads were pelleted and washed in NP‐40 lysis buffer. The supernatant was completely removed from the beads, and proteinase K buffer was added to the pelleted beads. Proteinase K was added at 0.5 mg mL^−1^ and incubated at 55 °C for 30 min to isolate immunoprecipitated RNA. The primers used for PCR are listed in Table S5 (Supporting Information).

### Dual‐Luciferase Reporter Assay

293T cells were cotransfected with each DUXAP9 promoter‐luciferase construct and pRL‐TK promoter Renilla luciferase construct in the vector group, wild‐type YY1 group and mutant YY1 group for 48 h. Luciferase assays were performed using a dual‐luciferase reporter assay kit (Beyotime, China) according to the manufacturer's instructions.

### Chromatin Immunoprecipitation (ChIP)

ChIP assays were performed on CAL27 cells with a SimpleChIP Enzymatic Chromatin IP Kit (#S9003, CST, USA) according to the manufacturer's instructions. IgG was used as the negative control, and an anti‐YY1 antibody (#46395S, CST, USA) and anti‐EZH2 antibody (#5246, CST, USA) were used to pull down the promoter regions of target genes. Then, ChIP DNA was analyzed by qPCR using specific primers, and the data were normalized to the input DNA. The results were derived from three independent experiments. The primers used for ChIP‐qPCR are listed in Tables S6 and S7 (Supporting Information).

### RNA Pull‐Down Assay and Liquid Chromatography Tandem Mass Spectrometry (LC‐MS/MS)

A biotinylated RNA pull‐down assay was conducted using a Pierce Magnetic RNA‐Protein Pull‐Down Kit (Thermo Scientific, USA) following the manufacturer's instructions. CAL27 cells were crosslinked in 1% formaldehyde for 10 min, equilibrated in glycine buffer for 5 min, washed with cold PBS three times, scraped with 1 mL of lysis buffer and incubated for 10 min. The cell samples were sonicated and then centrifuged, after which the supernatant was transferred to a 2 mL tube, and 50 µL was saved for input analysis. The lysate supernatant was incubated with DUXAP9 probes (RiboBio) or a negative probe for 3 h at room temperature with rotation; then, 100 µL of streptavidin magnetic beads was added, and the mixture was incubated for 1 h with stirring. The bead/sample mixture was washed twice, after which the mixture was subjected to protein purification. After subsequent washes, the pulled‐down complexes were separated by SDS‐PAGE, and the distinct protein bands were excised, dissolved, and subjected to LC‐MS/MS, which was performed by Shanghai Jiao Tong University School of Medicine Protein Technology Core.

### Coimmunoprecipitation (co‐IP)

For the co‐IP assay, cells were lysed with IP buffer (150 mm NaCl, 50 mm Tris‐HCl pH = 8.0, 1% Nonidet P‐40, 25 mm NaF, 2 mm Na3VO4, and 1% protease inhibitor cocktails and 5 mm PMSF before use) on ice for 30 min, and the cell lysate was harvested by centrifugation. The precleaning step was followed by incubation with the indicated antibodies and Protein A/G magnetic beads (B23202, Bimake, USA) at 4 °C overnight. The next day, the beads bound by target proteins were washed 6 times with IP buffer. Proteins were denatured at 95 °C for 10 min for WB analysis.

### Ubiquitination Assay

Cultured cells were treated with 20 µm MG132 (HY‐13259, MCE, USA) for 6 h and then lysed in IP lysis buffer containing protease and phosphatase inhibitors on ice for 30 min. Anti‐EZH2 antibody (#5246, CST, dilution 1:100) or IgG was added to the lysate and incubated with rotation overnight at 4 °C. Protein A/G magnetic beads (B23202, Bimake, USA) were added to the mixture, incubated at 4 °C for 2 h, boiled in SDS loading buffer, and used for western blotting analysis. Antiubiquitin antibody (#3936, CST, dilution 1:1000) or anti‐HA antibody (51064‐2‐AP, Proteintech, USA) was used to detect the ubiquitination of EZH2.

### Western Blot Analysis

Cells were collected at the indicated times in SDS lysis buffer (Beyotime, China). The protein concentration was determined by a BCA protein assay kit (Beyotime, China). Equal amounts of proteins were separated by sodium dodecyl sulfate‐polyacrylamide gel electrophoresis (SDS‐PAGE) and transferred to polyvinylidene difluoride membranes. The membrane was soaked in 10% skim milk in PBS for 1 h at room temperature and incubated with primary antibody overnight at 4 °C. Then, antimouse or antirabbit horseradish peroxidase (HRP)‐conjugated secondary antibodies (AS003/AS014, Abclonal, dilution 1:5000) were used. Afterward, the protein–antibody complex was visualized by enhanced chemiluminescence assay (SB‐WB012, Share‐bio, China). *β*‐Tubulin was used as a control. Primary antibodies against EZH2 (#5246, CST, USA, dilution 1:1000), phospho‐EZH2 (Thr345) (TA3584, Abmart, China, dilution 1:1000), phospho‐EZH2 (Thr487) (TA7400, Abmart, China, dilution 1:1000), N‐Cadherin (A3045, Abclonal, China, dilution 1:1000), E‐Cadherin (A11492, Abclonal, China, dilution 1:1000), Vimentin (A19607, Abclonal, China, dilution 1:1000), *β*‐Tubulin (A112289, Abclonal, China, dilution 1:1000), H3 (BS1174, Bioworld, China, dilution 1:1000), H3K27me3 (#9733, CST, USA, dilution 1:1000), YY1 (#46395S, CST, USA, dilution 1:1000), HA (51064‐2‐AP, Proteintech, USA, dilution 1:1000), FLAG (20543‐1‐AP, Proteintech, USA, dilution 1:1000), GAPDH (A19056, Abclonal, China, dilution 1:1000), CDK1 (A11420, Abclonal, China, dilution 1:1000) were used.

### Immunohistochemistry

Paraffin‐embedded tissue sections were dewaxed and rehydrated before antigen retrieval by boiling in 10 mm citrate buffer (pH = 6.0) for 30 min. Then, 3% hydrogen peroxide was added for 15 min to remove endogenous peroxidase. Tissues were incubated with goat serum for 30 min at room temperature and then with anti‐EZH2 (#5246, CST, USA, dilution 1:100), anti‐Ki67 (#GB111499, Servicebio, China, dilution 1:200), anti‐PCNA (A12427, Abclonal, China, dilution 1:100), and anti‐E‐cadherin (A11492, Abclonal, China, dilution 1:1000) antibodies at 4 °C overnight. Immunodetection was performed on the following day using DAB (Servicebio) according to the manufacturer's instructions. The staining scores were determined by two independent observers based on both the proportion and brown intensity of the indicated protein‐positive cells. The staining scores were determined by two independent observers based on both the proportion and brown intensity of the indicated protein‐positive cells using ImageJ. The proportion of positively stained tumor cells was divided into 4 grades: 0: no positive cells; 1: <10%; 2: 10–50%; and 3: >50%. The staining intensity was recorded as follows: 0 (no staining), 1 (light brown), 2 (brown), and 3 (dark brown). The SI was calculated as follows: SI = the proportion of positive cells × staining intensity × 10.

### Animal Experiment

The animal experiments were approved by the Shanghai Jiao Tong University School of Medicine laboratory animal care and use committee (SH9H‐2022‐A099‐SB). All animal experiments, implemented in BALB/C nude mice (6 weeks old) (Shanghai Laboratory Animal Center, Shanghai, China), were conducted in accordance with the appropriate ethical standards and national guidelines. DUXAP9, Vector, SS‐NC, or SS‐DUXAP9 cells (1 × 10^7^) were suspended in 0.1 mL of sterile PBS and subcutaneously injected into the left and right dorsal flanks of six mice. Four weeks later, all mice were euthanized, and tumor volumes were measured. All tumors were collected for IHC staining. For the metastasis assay, nude mice were injected with vector‐ or DUXAP9‐expressing CAL27 cells (2 × 10^6^) via the tail vein. For bioluminescence imaging, mice were injected with 150 µg g^−1^ D‐luciferin (Beyotime, China) 15 min before imaging. After 8 weeks, the mice were sacrificed, and the lungs were collected and fixed in 10% formalin. The number of metastatic colonies in the lungs was counted under a dissection microscope, and the lung tissue sections were prepared and stained with hematoxylin and eosin.

### Statistical Analysis

Statistical analysis was performed using R (3.6.1). Statistical differences were evaluated using two‐tailed unpaired Student's *t*‐test for comparisons between two groups. A *p* value of less than 0.05 (*p < 0.05, **p < 0.01, and ***p < 0.001) was considered statistically significant. Statistical methods and corresponding p values for data shown in each panel are included in the figure legends. Analyses of associations between DUXAP9 or YY1 levels and clinical features were performed using the Wilcoxon rank‐sum test. A Spearman correlation test was used to analyze the association between YY1 and DUXAP9 levels.

### Ethical Statement

All tissue samples used for the Sharing Platform were collected from the Department of Oral and Maxillofacial‐Head and Neck Oncology, Shanghai Ninth People's Hospital, Shanghai Jiao Tong University School of Medicine. The study was approved by the Ethics Committee of Shanghai Ninth People's Hospital affiliated with Shanghai Jiao Tong University, School of Medicine (2018‐86‐T77).

## Conflict of Interest

The authors declare no conflict of interest.

## Author Contributions

W.Z., Y.F., and C.L. contributed equally to this work. W.C. and X.Y.Z. conceived and designed the study. W.K.Z., Y.S.F., and C.K.C., conducted the experiments. S.Y.Z., Z.Q.H., and C.Z.L. analyzed the data. W.K.Z., W.C., and X.Y.Z. wrote the manuscript. J.Y.X. helped perform the analysis with constructive discussions. All authors have read and approved the final manuscript.

## Supporting information

Supporting Information

## Data Availability

The data that support the findings of this study are available in the supplementary material of this article.
