# Supplementary material for: Yin Yang 1‐Induced Long Noncoding RNA DUXAP9 Drives the Progression of Oral Squamous Cell Carcinoma by Blocking CDK1‐Mediated EZH2 Degradation
Source: Adv Sci (Weinh). 2023 Jul 3;10(25):2207549. doi: 10.1002/advs.202207549 (PMC10477890; doi:10.1002/advs.202207549)
Supplement: Supplementary file 1 — Supporting Information [file ADVS-10-2207549-s001.pdf]

ADVANCED SCIENCE
Open Access

Supporting Information

for *Adv. Sci.*, DOI 10.1002/advs.202207549

Yin Yang 1-Induced Long Noncoding RNA DUXAP9 Drives the Progression of Oral Squamous Cell Carcinoma by Blocking CDK1-Mediated EZH2 Degradation

Wenkai Zhou, Yisheng Feng, Chengzhong Lin, Chi Kuan CHAO, Ziqi He, Shiyao Zhao, Jieyuan Xue, Xu-Yun Zhao and Wei Cao**

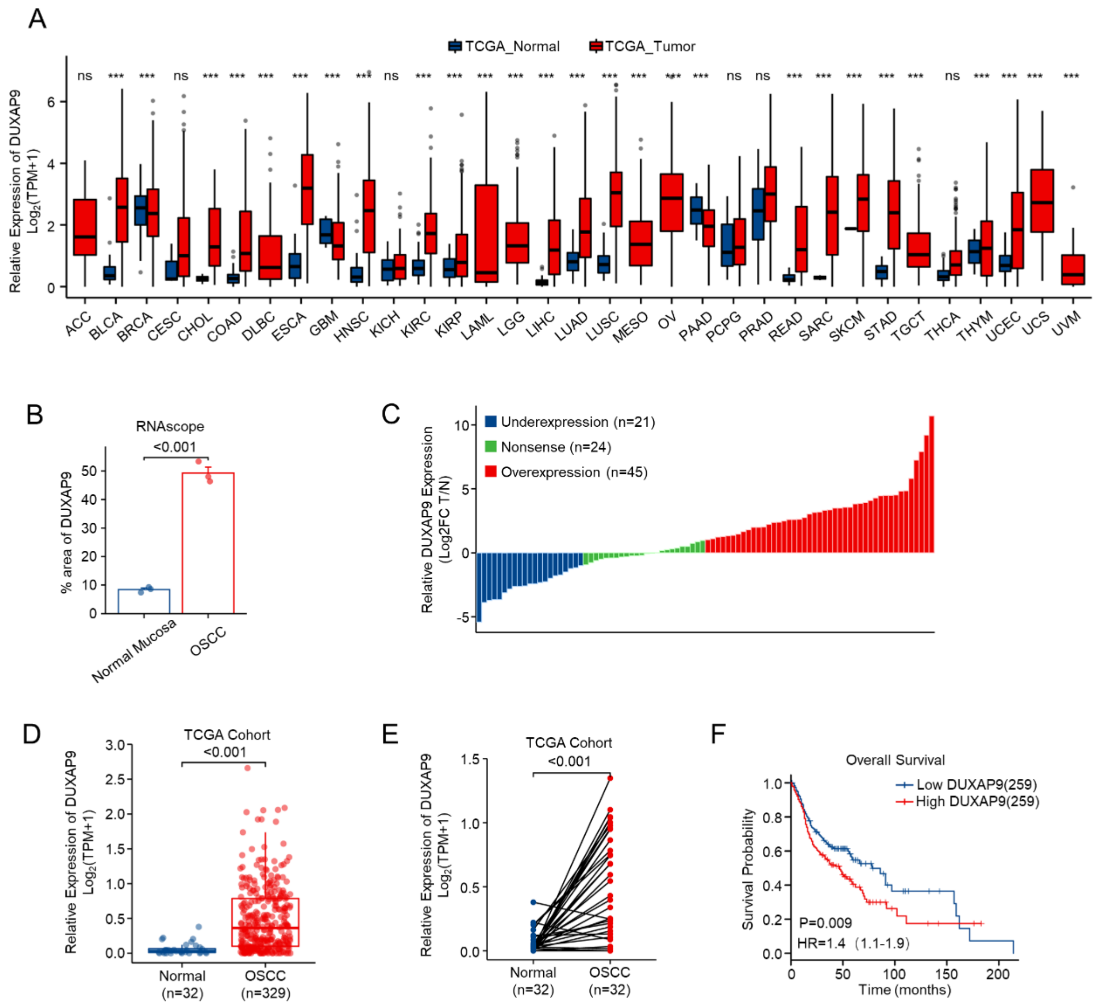

Figure S1 DUXAP9 is highly expressed in OSCC tissues and correlated with poor survival rates.

A) The expression of DUXAP9 in a set of cancer cohorts from the TCGA database. B) The expression of DUXAP9 in normal mucosa and OSCC by RNAscope assay. C) DUXAP9 expression from the SJTU cohort was classified into three groups according to the expression ratio (T/N) of OSCC tissues (T) to adjacent normal tissues (N). D) The expression of DUXAP9 in OSCC and normal tissues from the TCGA database. E) The expression of DUXAP9 in paired OSCC tissues from the TCGA database. F) Kaplan–Meier curves of the overall survival rate were generated using Gene Expression Profiling Interactive Analysis (GEPIA). Data are presented as the mean ± SD from three independent experiments. Data in A and D were calculated by the Wilcoxon rank sum test. Data in C were calculated by two-tailed unpaired Student’s t test. Data in E were calculated by the Wilcoxon signed rank test. Data in F were calculated by the log rank test.

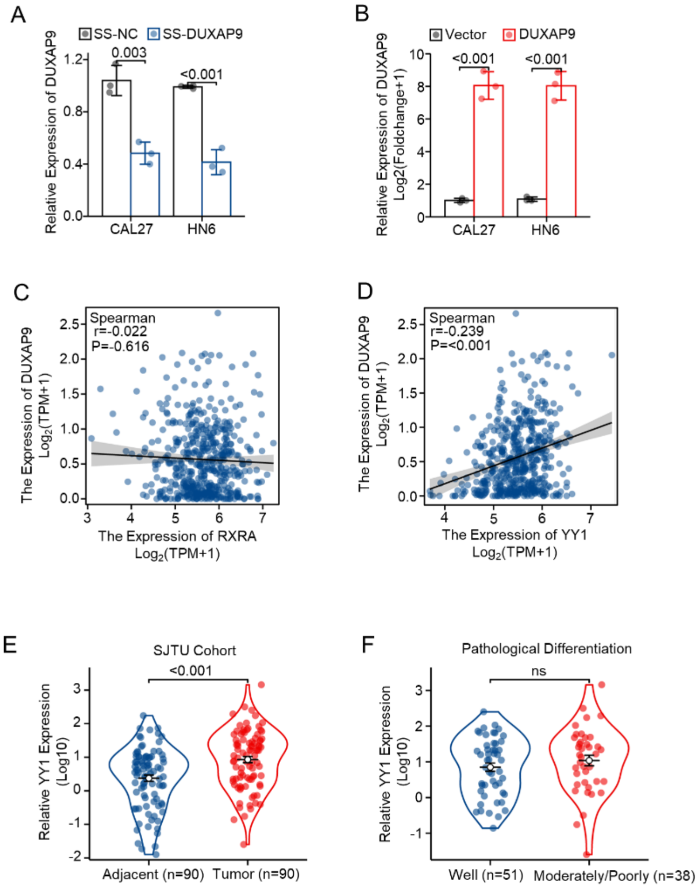

Figure S2. The expression of DUXAP9 in OSCC cells and the correlation between DUXAP9 and transcriptional factors and YY1 correlation with clinicopathologic features.

A, B) The expression of DUXAP9 in CAL27 and HN6 cells transfected with SS-NC, SS-DUXAP9 ASO (A) and control- and DUXAP9-expressing vectors (B) was measured by qRT-PCR. C, D) The correlation of mRNA expression between DUXAP9 and RXRA (C) or YY1 (D) using the TCGA database. E) The expression of YY1 in OSCC tissues (Tumor) and adjacent normal tissues (Adjacent) was measured by qRT-PCR. F) The expression of YY1 in OSCC tissues classified according to pathological differentiation. Data are presented as the mean ± SD from three independent experiments. Data in A and B were calculated by two-tailed unpaired Student’s t-test. Data in C and D were calculated by the Spearman correlation test. Data in E and F were calculated by the Wilcoxon rank-sum test.

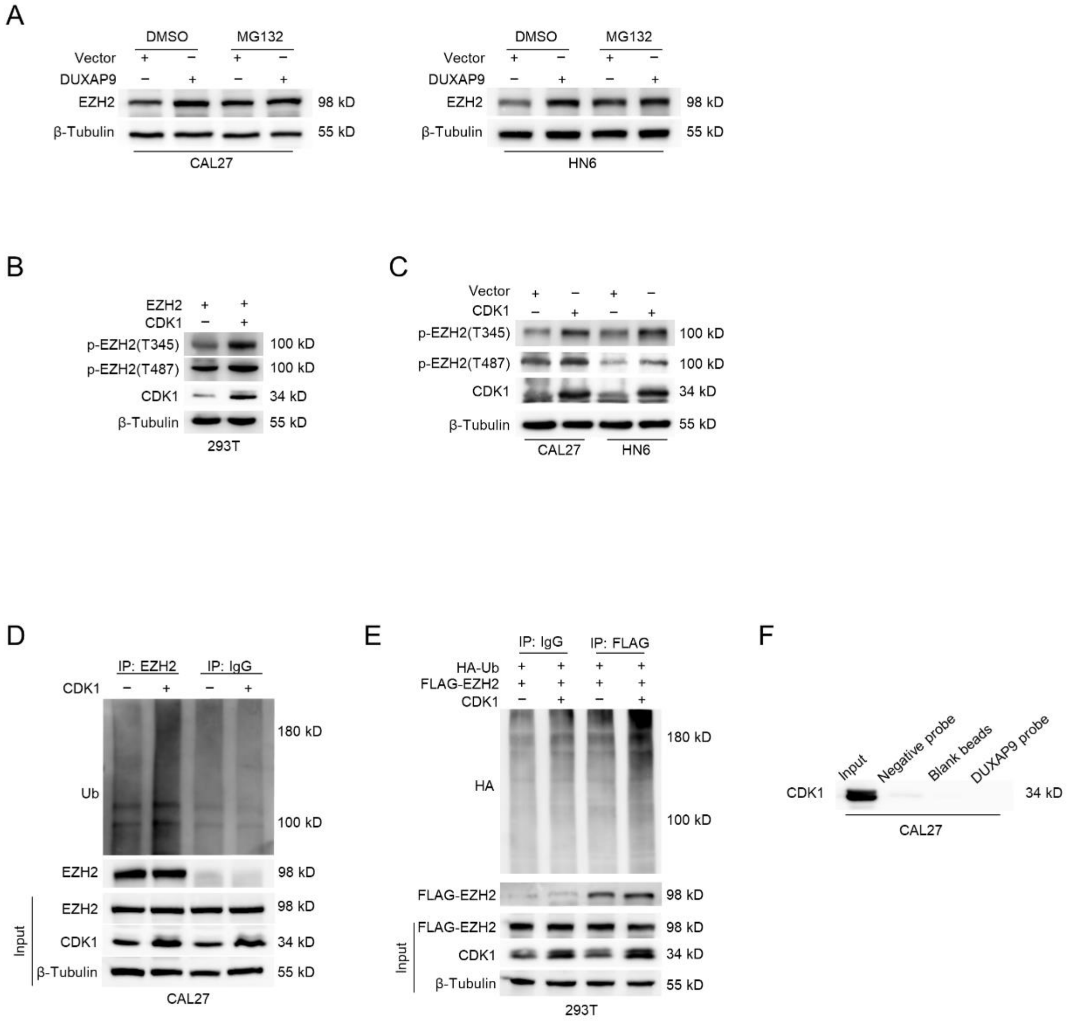

Figure S3 CDK1 induces the phosphorylation and ubiquitination of EZH2 and does not interact with DUXAP9.

A) Western blot shows EZH2 protein in CAL27 and HN6 cells transfected with control- or DUXAP9-expressing vectors followed by MG132 treatment (20 μM for 6 h). B, C) Western blot showing the expression of p-EZH2 (T345), p-EZH2 (T487), and CDK1 in CDK1-overexpressing 293T (B), CAL27 and HN6 (C) cells treated with MG132 (20 μM for 6 h). D, E) Western blot shows the ubiquitination of EZH2 in CAL27 (D) and 293T (E) cells transfected with CDK1-overexpressing vector and treated with MG132 (20 μM for 6 h). F) The binding of DUXAP9 with CDK1 was detected by RNA pull-down assay.

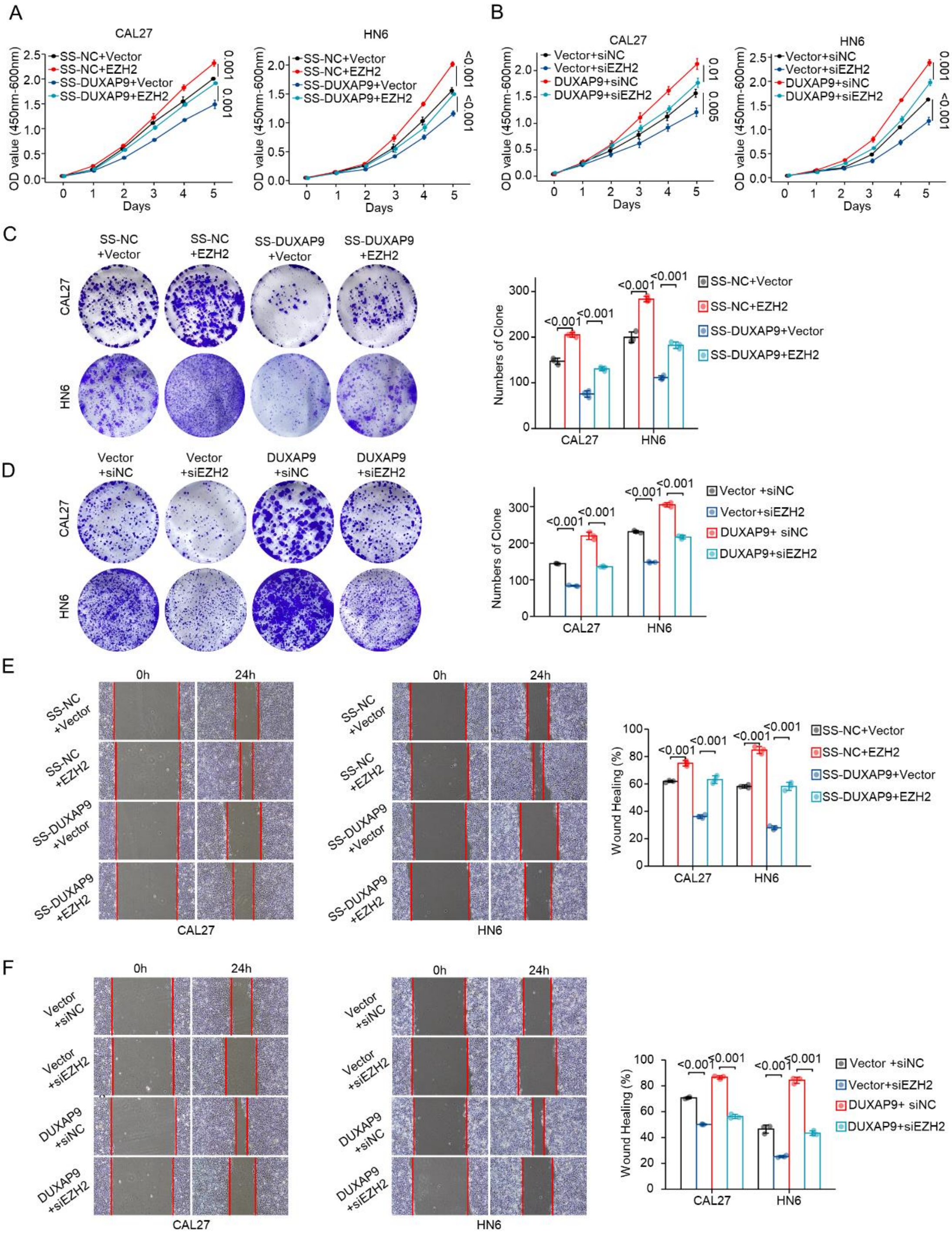

Figure S4. The expression of EZH2 is required for DUXAP9-induced proliferation and metastasis of OSCC cells.

A-F) Images of CCK-8 assays (A), colony-formation assays (C) and wound-healing assays (E) of CAL27 and HN6 cells co-transfected with SS-NC or SS-DUXAP9 ASO and control- or EZH2-expressing vectors in combination, n = 3/group. Images of CCK-8 assays (B), colony-formation assays (D) and wound-healing assays (F) of CAL27 and HN6 cells co-transfected with control or DUXAP9-expressing vectors and siRNAs targeting control (NC) or EZH2 in combination, n = 3/group. Data are presented as the mean ± SD from three

independent experiments. Data were calculated by two-tailed unpaired Student’s t test.

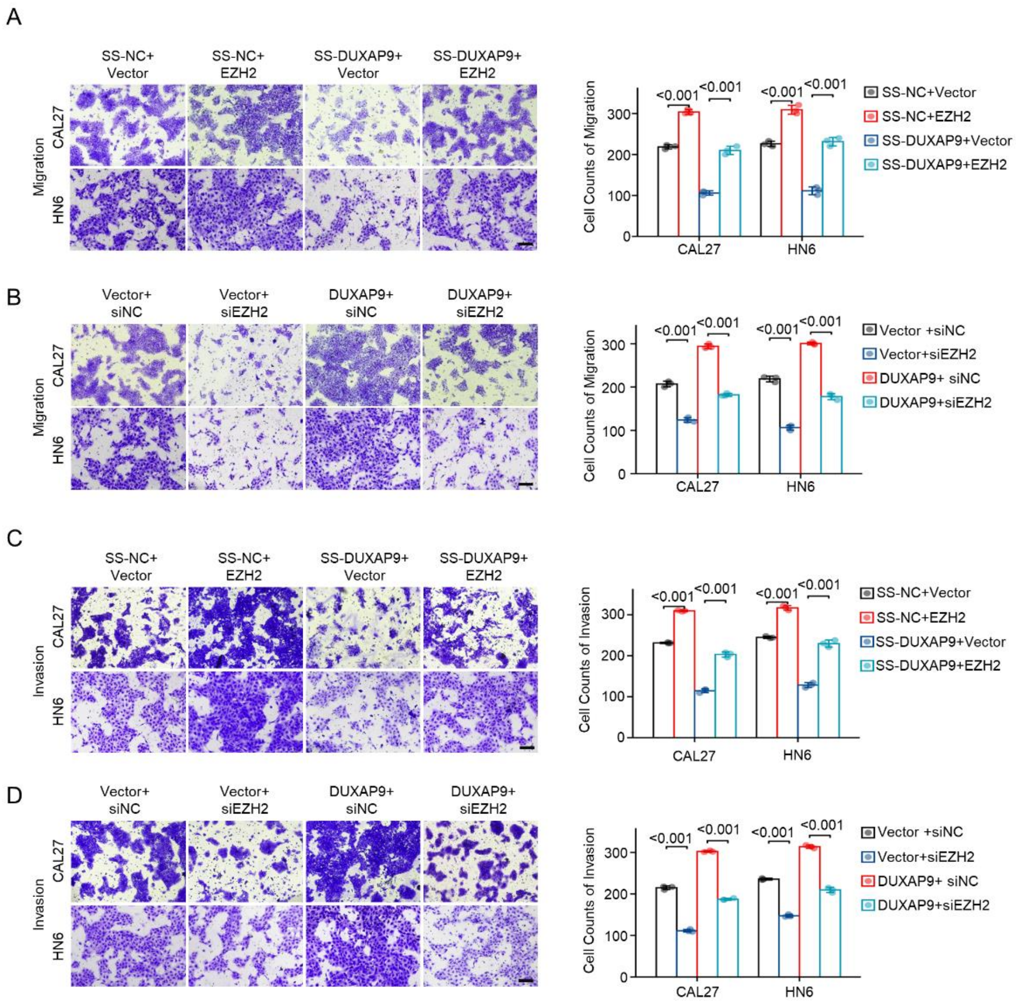

Figure S5. The expression of EZH2 is required for DUXAP9-induced metastasis and invasion of OSCC cells.

A, C) The migration (A) and invasion (C) behavior of CAL27 and HN6 cells transfected with SS-NC or SS-DUXAP9 ASO and control- or EZH2-expressing vectors in combination by transwell assays, n = 3/group. B, D) The migration (B) and invasion (D) behavior of CAL27 and HN6 cells transfected with control- or DUXAP9-expressing vectors and siRNAs targeting control (NC) or EZH2 in combination by Transwell assays, n = 3/group. Scale bars, 100 μm. Data are presented as the mean ± SD from three independent experiments. Data were calculated by two-tailed unpaired Student’s t test.

Table S1. The clinicopathological parameters of 5 HNSCC patients

Patient No.	Age	Gender	Smoker history	Alcohol history	Histologic grade	TNM stage	Anatomic site	Lymphovascular invasion
1	56	Female	No	No	I-II	T2N0M0	Gingiva	No
2	54	Male	Yes	Yes	I	T3N0M0	Gingiva	No
3	58	Male	No	No	III	T2N0M0	Tongue	Yes
4	64	Male	Yes	Yes	II	T2N0M0	Tongue	No
5	57	Female	No	No	III	T4aN0M0	Tongue	No

Table S2. Smart Silencer and siRNA targeting sequences.

**Product Name**	**Targeting Sequences**
DUXAP9 Smart Silencer	GCACACTGTTTCAACTCTC
	GCACTTCACTGTCACACTC
	GCAGCTGGGATTTGTAAGT
	CCATAGGTCACCAGCTTCAC
	GCTCAGCTTCCACACTCAGA
	GGAGCTCAAAGGATGCATTT
si-YY1	GCTGGACCACCTGATGAAT
si-EZH2*	GCTGAAGCCTCAATGTTTA

Table S3. Sequences of DUXAP9 probes.

**Primer No.**	**Sequences (5'-3')**
Probe_1	GTTTCCTTCCATGAACCTTT-/3bio/

Probe_2	TGTGTCTGTGAGCCAGCTTG-/3bio/
Probe_3	TGCTGAGGAGCAAGACATCC-/3bio/
Probe_4	CATACCTTGCTCAACTACTC-/3bio/
Probe_5	GTCAGCCACTGATCTGTTCC-/3bio/
Probe_6	CACCCTACCTTAGGAAACAG-/3bio/

Table S4. Sequences of qRT-PCR primers.

Primers		Sequences (5'-3')
DUXAP9	Forward	TGGCTGGTGGAGGATGTCTT
	Reverse	CCTGGGCTCCCTCAAATCAG
18s	Forward	CAGCCACCCGAGATTGAGCA
	Reverse	TAGTAGCGACGGGCGGTGT
U6	Forward	CTCGCTTCGGCAGCACATATACT
	Reverse	ATTTGCGTGTCATCCTTGCGCA
GAPDH	Forward	GAACGGGAAGCTCACTGG
	Reverse	GCCTGCTTCACCACCTTCT
NEAT1	Forward	GCATACGCAGCAGATCAGCAT
	Reverse	CCCACAATATAGGCATTTACAAGG
MALAT1	Forward	CCTAACCAGGCATAACACAGAAT
	Reverse	CGAATGGCTTTGTCTCCGAA
EZH2	Forward	GTACACGGGGATAGAGAATGTGG
	Reverse	GGTGGGCGGCTTTCTTTATCA
E-cadherin	Forward	CGAGAGCTACACGTTCACGG
	Reverse	GGGTGTCGAGGGAAAAATAGG
N-cadherin	Forward	TGCGGTACAGTGTAACTGGG

	Reverse	GAAACCGGGCTATCTGCTCG
Vimentin	Forward	AGTCCACTGAGTACCGGAGAC
	Reverse	CATTTCACGCATCTGGCGTTC

β-actin	Forward	GATGAGATTGGCATGGCTTT
	Reverse	GAGAAGTGGGGTGGCTT
DAB2IP	Forward	CTGAGCGGGATAAGTGGATGG
	Reverse	AAACATTGTCCGTCTTGAGCTT
CDKN1A	Forward	AGTCAGTTCCTTGTGGAGCC
	Reverse	CATTAGCGCATCACAGTCGC
CDKN2A	Forward	GATCCAGGTGGGTAGAAGGTC
	Reverse	CCCCTGCAAACTTCGTCCT
RUNX2	Forward	TGGTTACTGTCATGGCGGGTA
	Reverse	TCTCAGATCGTTGAACCTTGCTA
TCF7	Forward	CTGGCTTCTACTCCCTGACCT
	Reverse	ACCAGAACCTAGCATCAAGGA
MMP7	Forward	GAGTGAGCTACAGTGGGAACA
	Reverse	CTATGACGCGGGAGTTTAACAT
IL1R1	Forward	ATGAAATTGATGTTCGTCCCTGT
	Reverse	ACCACGCAATAGTAATGTCCTG
YY1	Forward	AAGAGCGGCAAGAAGAGTTAC
	Reverse	CAACCACTGTCTCATGGTCAATA
CSTA	Forward	AAACCCGCCACTCCAGAAAT
	Reverse	CATGCTGCTAAAAGCCCGTC
SLUG	Forward	CGAACTGGACACACATACAGTG
	Reverse	CTGAGGATCTCTGGTTGTGGT
TWIST	Forward	TGCCAGGACCGCTTCTAC

	Reverse	CACCTGGTTCAACTCACTCC
SNAIL	Forward	CCCCAATCGGAAGCCTAACT
	Reverse	CGTAGGGCTGCTGGAAGGTA

Table S5. Sequences of RIP primers for DUXAP9.

**Primer No.**		**Sequences (5'-3')**
P1	Forward	CCCTGCAGCAGCAGCGG
	Reverse	GCGGAGACCTGCCTCCTAC
P2	Forward	GGTGGAGTAGGAGGCAGGTC
	Reverse	TTCAAAGTAGCTGTTCATCCACAGA
P3	Forward	TCTCTTTATCTGTGGATGAACAGCT
	Reverse	GGGAAGTGGAGGCAGTCTCA
P4	Forward	TCAAACACAGCTGCAGGGATG
	Reverse	GTACAGCAGTTTCCTCATCCCT
P5	Forward	GTACAGCAGTTTCCTCATCCCT
	Reverse	GCGAGACTGCCCATCCACA
P6	Forward	CATCTGTGGATGGGCAGTCTC
	Reverse	TTCTTTCCTGGGCGTGGTGG
P7	Forward	CTGGGATTACAGGCACCCG
	Reverse	CATCCTCCACCAGCCAGTTG
P8	Forward	CCAGGAGCCCCAACTGGC
	Reverse	GGGGTGCAGGAAGGGGAAG
P9	Forward	CCCTTCCCATAGGTCACCAGC
	Reverse	GCTGAGCCCAGGGCAGAT
P10	Forward	TTTGTCCATCTGCCCTGGGC
	Reverse	CACAGGTGAGCTGTGGCG

P11	Forward	CTCACATCCCTCCACCGTCG
	Reverse	AAACAGTGTGCGTTTGGGGT
P12	Forward	CACGGCCAAGGTCACACT
	Reverse	GGTGTGAGTGTGTGAGCATCC
P13	Forward	AGCCACATGGATGCTCACAC
	Reverse	ACACTCACACACACTCCCACC

Table S6 Sequences of ChIP primers for the promoters of DUXAP9.

**Primer No.**		**Sequences (5'-3')**
NC	Forward	GCTGAATCTCTGAAGAGACCG
	Reverse	TCACCAGGGCTGCCTGGGTCA
motif 1	Forward	CAGACTGCTCCCTTCACCTT
	Reverse	GGTGGAGAGTTCTGTAGATG
motif 2	Forward	CTTTAAGAGGGGGTGGAGCT
	Reverse	TCCACGCCCCCGCCACTTA

Table S7 Sequences of ChIP primers for the promoters of PRC2 complex targets.

**Primer No.**		**Sequences (5'-3')**
CDKN1A	Forward	TTTTGTCCTTGGGCTGCCTG
	Reverse	GCAGATCACATACCCTGTTCAG
RUNX2	Forward	CGTAGTAGTACACAACGCCG
	Reverse	GTTTCGTGTCTGTCTTCCCC
DAB2IP	Forward	CCTGCTTTCTGTTTCCTTCTCCTG
	Reverse	TTGAACCACCTCCTCCTCCCTCTC

Table S8 The number of samples in Figure S1A.

X	Group	N
ACC	Tumor	79
BLCA	Normal	19
BLCA	Tumor	412
BRCA	Normal	113
BRCA	Tumor	1113
CESC	Normal	3
CESC	Tumor	306
CHOL	Normal	9
CHOL	Tumor	35
COAD	Normal	41
COAD	Tumor	480
DLBC	Tumor	48
ESCA	Normal	11
ESCA	Tumor	163
GBM	Normal	5
GBM	Tumor	169
HNSC	Normal	44
HNSC	Tumor	504
KICH	Normal	25
KICH	Tumor	65
KIRC	Normal	72
KIRC	Tumor	541
KIRP	Normal	32
KIRP	Tumor	291
LAML	Tumor	150
LGG	Tumor	532
LIHC	Normal	50
LIHC	Tumor	374
LUAD	Normal	59
LUAD	Tumor	539
LUSC	Normal	49
LUSC	Tumor	502
MESO	Tumor	87
OV	Tumor	381
PAAD	Normal	4
PAAD	Tumor	179
PCPG	Normal	3
PCPG	Tumor	184
PRAD	Normal	52
PRAD	Tumor	501

READ	Normal	10
READ	Tumor	167
SARC	Normal	2
SARC	Tumor	263
SKCM	Normal	1
SKCM	Tumor	472
STAD	Normal	32
STAD	Tumor	375
TGCT	Tumor	156
THCA	Normal	59
THCA	Tumor	512
THYM	Normal	2
THYM	Tumor	120
UCEC	Normal	35
UCEC	Tumor	554
UCS	Tumor	57
UVM	Tumor	80
